# Communication Processes Related to Decision‐Making in Medication Management Between Healthcare Providers, Older People and Their Carers: A Systematic Review

**DOI:** 10.1111/hex.70252

**Published:** 2025-04-20

**Authors:** Deana M. Copley, Elizabeth Manias, Vanessa Watkins, Alison M. Hutchinson

**Affiliations:** ^1^ School of Nursing and Midwifery Deakin University Geelong Australia; ^2^ Centre for Quality and Patient Safety, Institute for Health Transformation Deakin University Geelong Australia; ^3^ School of Nursing and Midwifery Monash University Clayton Australia; ^4^ Barwon Health Geelong Australia

**Keywords:** aged, medication safety, medication therapy management, medication‐related decision‐making, older person, patient participation, shared decision‐making

## Abstract

**Objective:**

To examine decision‐making between healthcare providers (HCPs), older people and their carers in relation to medication management.

**Methods:**

Four databases were systematically searched up to June 2023. Two authors screened the search results. Extracted quantitative data were analysed descriptively, and qualitative data were analysed thematically.

**Results:**

Fifty‐three papers reporting on 49 studies were included. A variety of research methods were utilised. Few authors provided a definition of shared decision‐making (SDM). Three major themes were identified: provider‐driven decision‐making, patient‐driven decision‐making and a shared role in decision‐making. Some older people preferred or deferred to provider‐driven decision‐making, mainly due to trust in the HCP's expertise. Other reasons for provider‐driven decision‐making were patient anxiety, declining health, lack of medical knowledge or poor communication during the clinical encounter. Evidence of patient‐driven decision‐making was often prompted by concerns about the adverse effects of medication. Most older people preferred or adopted a shared role in decision‐making.

**Conclusion:**

Whilst most patients and carers preferred to engage in SDM related to medication management, at times, they felt unable to do so, deferring to provider‐driven decision‐making. There is a need for a standardised definition and measurement of SDM.

**Patient or Public Contribution:**

This systematic review did not directly involve older people or carers of older people in the design or conduct of the review. However, the findings will inform a qualitative study aimed at exploring older people and their carers' experiences of medication‐related decision‐making in collaboration with their healthcare provider.

**Trial Registration:** CRD42019124862.

## 1 Introduction

Shared decision‐making (SDM) involves the patient partnering with their healthcare provider (HCP) to make mutually agreed healthcare decisions [1, 2, 3]. An effective patient–HCP relationship is associated with enhanced communication, where the HCP listens to patient concerns and preferences and provides information, and the patient can ask questions and express their preferences, resulting in collaborative decisions [3, 4, 5].

SDM has been embedded in health policy internationally [6], but the challenge remains to implement SDM, engaging both the HCP and the patient in the process [6, 7]. In a cross‐sectional survey study investigating HCPs' preferred and usual roles in SDM, HCPs reported that they found it challenging to implement SDM in their daily practice, often reverting to a more paternalistic communication style [8]. From a patient's perspective, the definition of SDM was explored using semi‐structured interviews, and the description of a ‘shared’ decision ranged from no interactive exchange to full patient engagement [9]. Authors of a systematic review examining the definitions of SDM found that the most commonly used definitions suggest a process whereby an HCP speaks and a patient and/or carer mostly listens and speaks when invited [10]. The authors highlighted the lack of consistency in conceptualising SDM [10], which may lead to variability when conducting and analysing research in the area.

As a person ages, the likelihood of developing chronic conditions increases [11, 12, 13]. Medications are a common treatment modality for managing chronic conditions [14], with approximately 60% of people aged 65 years and over, taking three or more medications and about 12% taking more than 10 medications daily [15, 16]. Managing multiple chronic conditions can be challenging for older people, who may struggle to understand healthcare treatments. They may also need advice from multiple HCPs and lack knowledge about their medications [17, 18, 19]. Further, they may decide for themselves which medication to consume based on various factors, including cost, adverse effects, whether it makes them feel better or acquisition of information about the medication [20, 21, 22]. Older people are also less likely to ask questions or seek clarification about their medications [23]. This may relate to patient presumptions, such as a perceived lack of time when visiting their HCP and the perception that a passive patient is a ‘good’ patient [23].

Medication‐related DM is a complex process and is even more complex for older persons with chronic conditions. Such people are at increased risk of uncertainties about the benefits of treatments, potentially inappropriate medication prescribing and adverse drug reactions [24, 25, 26]. SDM is considered ideal for older people requiring multiple medications for chronic health conditions [15], and the long‐term nature of chronic conditions requires patients to take an active role in their own management.

Carers are an integral component of medication management for the older person, particularly for those living at home with cognitive impairment or increasing frailty [26, 27, 28]. Carers assist the older person in many ways with their medications, including administration, recognising medication effects and clarifying information [29]. As such, carers should be involved in medication‐related DM discussions.

Effective communication is a multi‐dimensional concept and process [30] and requires careful deliberation and collaboration between the HCP and patient and/or carer, where risks need to be weighed against the benefits when making decisions about medication options [1, 7]. There is a growing body of literature related to engaging the older person and their carer in medication‐related decisions [29, 31]; however, to date, there has been no systematic review exploring the older person and their carer's perspectives of actual DM related to medication management. Therefore, the aim of this systematic review was to identify and synthesise the evidence examining DM between HCPs and older people and their carers in relation to medication management.

## 2 Methods

### 2.1 Design

A systematic review of the literature was undertaken. The review protocol was developed a priori and registered with PROSPERO (CRD42019124862). The PRISMA 2020 statement guided the reporting of the systematic review [32].

### 2.2 Search Process

Literature was systematically searched using EMBASE, Medline Complete, CINAHL Complete and APA PsycInfo from the databases' inception to November 2024. We used four databases in our search. Bramer et al. [33] found the use of EMBASE and Medline had the highest search yield. The authors also found that for domain‐specific reviews, databases like CINAHL (for nursing) and PsycINFO (for mental health) were appropriate for ensuring adequate coverage of the literature. Search terms were identified through an initial exploratory search and with assistance from a research librarian. Subject headings and synonyms related to patient–health professional communication specifically linked to SDM in medication management were used. Search terms were adapted to meet each database's requirements and combined using Boolean operators, AND and OR. The search strategy for the Medline Complete search is provided (Appendix A). All search results were exported into EndNote 21, where duplicates were removed. Reference lists of all included articles and related systematic reviews were examined to identify any additional potentially eligible studies.

### 2.3 Eligibility

Peer‐reviewed research publications were included if they addressed the perspectives of older adults (65 years and older) regarding decision‐making about their medications. Studies investigating carer and HCP perspectives of older adults' involvement in DM related to medications were also included. No limitations were applied to the types of health conditions for which medications were prescribed. All research designs and methodologies were considered for inclusion.

Studies conducted in aged care settings were excluded as residents generally do not self‐manage their medications and are reliant on an HCP for medication administration [34]. Otherwise, there were no restrictions on the setting (whether during a clinic visit or as an inpatient). A carer was defined as a relative or friend who assisted the patient with their medications. An HCP was defined as a practitioner, such as a doctor, pharmacist or nurse, who managed medications. Studies involving populations including and extending beyond the age group of interest for this systematic review were excluded if researchers did not report separate findings by age for the group of interest. The search was limited to English‐language publications.

### 2.4 Study Selection

Rayyan systematic review software [35] was used to screen the references against the eligibility criteria to identify potentially eligible publications. Two authors (D.C. and V.W. or E.M. and A.M.H.) independently reviewed titles and abstracts. Any study identified as relevant was retrieved in full‐text and assessed independently by two authors (D.C. and V.W. or E.M. and A.M.H.) for final inclusion in the review. All discrepancies in the selection process were resolved through discussion between the reviewers until consensus was achieved.

### 2.5 Data Extraction

An investigator‐developed Microsoft Excel spreadsheet was used for data extraction. Extracted data included the authors' names, publication year, country of study, research aims and design, setting, sample characteristics (size, gender, age, health condition, medications and socio‐demographic characteristics), details about data collection and description of analysis and results. Data were extracted by one reviewer (D.C.) and independently verified for accuracy by another reviewer (E.M. or A.M.H.).

### 2.6 Quality Appraisal

Each included publication was independently assessed for quality by two reviewers. The framework designed by Caldwell et al. [36] was used for the quality assessment as it evaluates the quality of quantitative, qualitative and mixed‐methods health research. The framework uses three possible answers (yes, partly and no) for 18 questions. A ‘partly’ score meant that limited information was provided. Discrepancies were resolved through discussion. No papers were excluded based on methodological quality.

### 2.7 Data Synthesis

A narrative synthesis of the quantitative and qualitative data was conducted. NVivo 12 was used to manage the extracted quantitative and qualitative data, as well as the coding of excerpts from the selected publications. The codes were then categorised into sub‐themes and themes [37]. Meta‐analysis of quantitative data could not be undertaken due to the divergent methodologies and clinical diversity across the studies. A meta‐analysis should only be considered if the included studies are sufficiently homogeneous to provide a meaningful summary [38]. If there is substantial heterogeneity in the included studies, a narrative synthesis is appropriate [38].

## 3 Results

**Figure 1:**
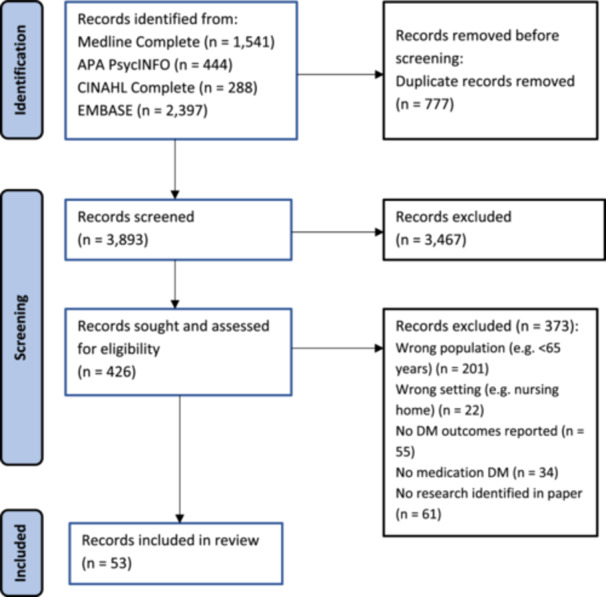
PRISMA flow diagram.

The database search yielded 4670 potentially eligible papers. After removing duplicates, the titles and abstracts of 3893 papers were reviewed. Overall, 3467 papers were excluded at this stage, leaving 426 full‐text papers for screening. From these, 53 papers reporting 49 studies met the inclusion criteria. Seven papers relating to three studies were included, as each paper reported a different aspect of the respective study [39, 40, 41, 42, 43, 44, 45]. Figure 1 shows the PRISMA flow chart of the search and study selection process.

### 3.1 Quality Assessment

The included papers were assessed using Caldwell et al.'s framework for critiquing health research [36]. Quality assessment results for included papers are reported in Appendix B.

### 3.2 Study Characteristics

The publication years of included papers ranged from 1994 to 2024, with over 50% (*n* = 32) published in the last 5 years. Most studies were undertaken in the United States (*n* = 15), Australia (*n* = 10), the United Kingdom (*n* = 7) and Canada (*n* = 5). There were 9 quantitative studies, 36 qualitative studies and 4 mixed‐methods studies. A variety of research methods were used, with all studies using either interviews and/or focus groups (*n* = 30), questionnaires (*n* = 9) or video/audio recordings of clinic visits (*n* = 3) or, as in a further seven studies, a combination of a questionnaire and an observational tool related to SDM were used [28, 39, 46, 47, 48, 49, 50]. Most interview guides and questionnaires were investigator‐designed and were heterogeneous in nature. In total, nine validated tools were used. Table 1 provides a summary of the studies.

**Table 1:** Summary of Studies.

	Year				Country						Method			Data Collection					Education level (%)			Decision Making (%)		
Author (s), (Year), [Ref]	Pre‐2000	2000‐2010	2011‐2020	2021+	USA	Australia	UK	Europe	Canada	China	Quantitative	Qualitative	Mixed	Interview &/or focus group	Survey &/or questionnaire	Video of clinic visits	Audio of clinic visits	Observation	≤ high school	> high school	Not reported	Provider driven	Shared	Patient driven
																			✓= reported narratively, % not provided					
Andreas et al. (2010) [51]		✓			✓							✓		✓							✗	✓	✓	
Belcher et al. (2006) [15]		✓			✓							✓		✓					35.3%	64.7%		✓	✓	
Bell et al. (2017) [70]			✓					✓				✓		✓							✗	✓		✓
Brünn et al. (2021) [58]^‡^				✓				✓				✓		✓							✗	✓	✓	
Campbell et al. (2020) [88]			✓						✓			✓		✓					60.4%	39.6%			✓	
Caughey et al. (2020) [71]			✓			✓							✓	✓	✓				87%	13%		40%	60%	
Dooley et al. (2019) [86]			✓				✓					✓				✓					✗		✓	
Eassey et al. (2017) [56]^‡^			✓			✓						✓			✓				36.6%	63.4%		✓	✓	
Fabricius et al. (2021) [52]			✓					✓				✓		✓				✓			✗		✓	
Farrell et al. (2020) [64]			✓						✓			✓		✓							✗	✓	✓	
Fried et al. (2017) [39] Mecca et al. (2022) [43]			✓	✓	✓						✓(RCT)		✓	✓			✓		68%	32%			✓	
Gillespie et al. (2019; 2022; 2023) [40, 41, 42]			✓	✓		✓							✓	✓	✓				46.7%	51.1%		15.3%	71.6%	
Green et al. (2020) [87]			✓		✓							✓					✓		51.6%	48.4%			✓	
Haverhals et al. (2011) [65]			✓		✓							✓	✓	✓							✗	✓	✓	✓
Højgaard et al. (2024) [78]				✓				✓				✓		✓							✗	✓	✓	✓
Holmqvist et al. (2019) [59]			✓					✓				✓	✓	✓							✗	✓	✓	
Hu et al. (2023) [55]				✓						✓	✓								75.5%	24.5%#		59.3%	35.4%	5.3%
Jansen et al. (2019) [53]			✓			✓						✓	✓	✓					73.3%	26.7%		✓	✓	✓
Junius‐Walker et al. (2021) [63]				✓				✓			✓				✓						✗	49%	49%	2%
Kempen et al. (2020) [80]			✓					✓				✓		✓							✗	✓	✓	✓
Knight et al. (2013) [79]			✓				✓					✓		✓							✗	✓		
Kreling et al. (2006) [66]		✓			✓							✓		✓							✗	✓		
Lansbury (2000) [62]		✓				✓						✓		✓							✗	✓		
Manias et al. (2024) [50]				✓		✓						✓	✓	✓				✓			✗	✓	✓	
McCabe et al. (2019) [28]			✓				✓					✓			✓	✓			61%	39%		✓	✓	
Mc Gillicuddy et al. (2019) [67]			✓				✓					✓		✓							✗	✓	✓	✓
O'Quinn et al. (2015) [82]			✓		✓							✓		✓					66.7%	33.3%		✓	✓	
Ouellet et al. (2022) [72]				✓					✓			✓		✓							✗	✓	✓	
Parekh et al. (2019) [81]			✓				✓					✓		✓							✗	✓		
Peat et al. (2023) [57]				✓			✓					✓		✓							✗	✓	✓	
Perreira, et al. (2022) [44, 45]				✓				✓				✓		✓							✗	✓		
Reeve et al. (2016) [73]			✓			✓						✓		✓							✗	✓	✓	
Ross & Gillett (2021) [74]				✓					✓			✓		✓							✗	✓		
Sale et al. (2011) [75]			✓						✓			✓		✓							✗	✓	✓	✓
Salter et al. (2014) [83]			✓				✓					✓		✓							✗		✓	✓
Schmittdiel et al. (2010) [85]		✓			✓						✓				✓				79%	21%			✓	
Schopf et al. (2018) [68]			✓					✓				✓		✓					66.7%	33.3%		✓	✓	
Smith et al. (1994) [60]	✓				✓						✓				✓				✓	✓		✓	✓	
Spinewine et al. (2005) [76]		✓						✓				✓		✓				✓			✗	✓	✓	
Thevelin et al. (2022) [49]				✓				✓					✓	✓	✓				62.5%	37.5%		77%*	23%*	✓
Tietbohl & Bergen (2022) [77]				✓	✓							✓				✓					✗	✓	✓	
Tinetti et al. (2024) [61]				✓	✓						✓(Non‐RCT)				✓				26.5%	73.5%			✓	
Tinetti et al. (2019) [47]			✓		✓						✓(Non‐RCT)				✓				53.4%	46.6%			✓	
Tjia et al. (2008) [69]		✓			✓							✓		✓					77.3%	22.7%		✓	✓	✓
Tobiano et al. (2021) [27]				✓		✓							✓	✓	✓			✓	83.7%	16.2%		29.6%	33.8%	2.8%
Weir et al. (2021) [54]				✓		✓						✓		✓							✗	✓	✓	
Weir et al. (2018) [46]			✓			✓						✓		✓					66.7%	33.3%		20%	✓	✓
Wilson et al. (2007) [84]		✓			✓						✓				✓				25.2%	74.8%			68.2%	
Xu et al. (2003) [48]		✓			✓						✓				✓				57.2%	42.8%			✓	

✓Information provided

✗No information provided

^$^ Study used mixed methods analysis of RCT data

^#^ Related to high school education and above

* Related to Interview data only

Seven papers in this review included a definition of SDM [15, 28, 51, 52, 53, 54, 55]. Other definitions related to SDM included two definitions of patient engagement [50, 56], three definitions of patient‐centred care [47, 54, 57] and one definition of risk communication [51]. Belcher et al. [15] identified that patient participants lacked an understanding of SDM, and the term was subsequently avoided in the conduct of interviews. Instead, the researchers asked participants about their perceptions of medication‐related decisions and how they might be a part of the process to help the doctor in choosing appropriate medications. A similar approach was used in other studies where researchers asked patients about what occurred in their last medical visit when medications were discussed and regimens changed and to describe how they were involved in the discussion [27, 49, 50, 58, 59]. In other studies, the emphasis of interview/survey questions and tools was on what HCPs said or asked and how the HCP made medication decisions [28, 46, 47, 48, 53, 54, 60, 61].

The combined sample across all studies comprised 23,932 participants. Of these, 296 were carers, 451 were HCPs and the rest were patients. Most studies (*n* = 44) were conducted in the community (participants were interviewed at home or in various community centres and online forums) and primary care settings (participants were sought from local medical practices and hospital outpatient clinics), with five studies conducted in a hospital inpatient setting. Table 2 provides details of the study characteristics.

**Table 2:** Study characteristics.

						Sample characteristics		
Author (s), (year), Country, ref.	Purpose/objectives	Design/method/theoretical framework	Instrument/measures related to SDM/instrument validation	Setting	*n* Response rate (%)	Older person age (mean [SD or range]) male gender (%)	Socio‐demographics	Health condition/medication
Andreas et al. (2010) The United States [51]	‘The goal of this study was to explore specific ways by which patients construct and communicate their understandings and orientations to risk’	Design—Exploratory Method—Qualitative study using focus groups Framework—Narrative theory	Instrument—Author‐designed interview guide Validation—Not reported	Primary care	*n* = 19	Age 73 (mean) Male gender—73.7%	Not reported	CVD/cardioprotective medications
Belcher et al. (2006) The United States [15]	This study ‘explored the perceptions of older adults regarding patient involvement in medication decision making’	Design—Exploratory Method—Qualitative study using semi‐structured interviews Framework—Not reported	Instrument—Author‐designed interview guide Validation—Pilot tested	Community primary care	*n* = 51 Response rate—Not reported	Age 77 (6.6) Male gender—37.3%	78% Caucasian; 20% African descent 39% married; 49% widowed; rest single. 88% retired. 35.3% of participants had secondary education or less	CVD, cancer, diabetes, arthritis ≥ 1 prescription medication
Bell et al. (2017) Norway [70]	‘To explore how home‐dwelling elderly FRID users perceive their fall risk and how they relate this to their drug use’	Design—Exploratory Method—Qualitative study using semi‐structured interviews Framework—Not reported	Instrument—Author‐designed interview guide Validation—Not reported	Community	*n* = 14	Age 81 (66–97) Male gender—50%	Not reported	≥ 2 prescription medications (related to fall‐risk increasing drug) *n* = 4 reported falls *n* = 4 reported dizziness
Brünn et al. (2021) Germany [58]^d^	‘To explore patients' attitudes and beliefs to coping with polypharmacy, their experiences of integrating polypharmacy into their daily lives, their views on GPs' use of digital interventions to support medication management’	Design—Exploratory Method—Qualitative study using semi‐structured interviews Framework—Not reported	Instrument—Author‐designed interview guide Validation—Pilot tested	Community	*N* = 14 *n* = 8 (aged ≥ 65 years)	Age 56–88 years^d^ Male gender—25% ( > 65 years)	25% lived in a large city and alone 75% lived in a medium‐sized city with a spouse	≥ 5 prescription medications
Campbell et al. (2020) Canada [88]	‘The objective was to assess the implementation of (the) interventions and learn how to better support behaviour change for CV risk reduction in order to make further modifications to the interventions (targeting finance and knowledge) for the rest of the trial’	Design—Exploratory Method—Qualitative study using semi‐structured telephone interviews and focus groups Framework—Health Belief Model	Instrument—Author‐designed interview guides Validation—Pilot tested for flow	Community	*n* = 53 consumers *n* = 20 pharmacists Response rate = 50.6% consumers and 74.1% pharmacists	Age > 65 years Male gender (consumer)—56.6% Male gender (pharmacist)—55%	All consumers were of low socio‐economic status 83% native English speakers 36% lived in a single‐person household.	High risk of CV events (kidney disease, heart failure, hypertension, diabetes), taking medication
Caughey et al. (2020) Australia [71]	‘This study aimed to examine how older patients with multimorbidity balance the benefits and harms associated with a medication, and in the presence of competing health outcomes’	Design—Exploratory descriptive Method—Mixed Quantitative study (Risk Ratio hypotheticals) and Qualitative study (semi‐structured interviews) Framework—Not reported	Instrument—Author‐designed interview guides Validation—Not reported	Primary care	*n* = 15 patients *n* = 5 clinicians Response rate—Not reported	Age 79 (73–86) Male gender—47%	87% of patients had secondary education or less. 73% married; rest single/divorced (20%), widowed (7%)	≥ 2 chronic health conditions taking medication
Dooley et al. (2019) The United Kingdom [86]	‘The aim of this study was to examine how doctors involve patients with dementia in decisions to start medication…and how this affects patient acceptance of medication’	Design—Exploratory Method—Qualitative study using video recordings of clinic visits Framework—Not reported	Instrument ‐ Treatment Recommendation Coding Scheme Validation—Previously validated	Primary care	*n* = 71 patients *n* = 21 HCPs	Age 81 (65–91) Male gender (patient)—38% Male gender (HCP)—47.6%	Companion present for 67 patients 83.4% family members 87.3% Caucasian	69% Alzheimer's disease 39.4% dementia types
Eassey et al. (2017) Australia [56]^d^	The ‘aim of this project was to gather patient insights and experiences to develop and investigate multidisciplinary integrative strategies that will support patients in safely navigating the health system and avoiding medication‐related problems’	Design—Cross‐sectional study Method—Qualitative study using a self‐report online survey Framework—Not reported	Instrument—Author‐designed survey Validation—Not reported	Community	*n* = 506 Response rate—Not reported	Age 64 (8.2)^d^ Male gender—38.5%	93.3% Caucasian 55% live in metropolitan areas 45% in rural areas 31.2% had trade and 32.2% had university education	≥ 5 prescription medications, discharged from hospital to home in previous month
Fabricius et al. (2021) Denmark [52]	The ‘study was to explore the determinants of patient involvement in decisions made in the emergency department about the patient's medication’	Design—Ethnography Method—Qualitative study using observation followed by semi‐structured interviews Framework—Formalia framework (‘Confidential space’)	Instrument—Author‐designed observation guide Validation—Not reported Instrument—Author‐designed interview guide Validation—Pilot tested	Hospital inpatient	*n* = 68 HCPs	Age ≥ 75 years Male gender—Not reported	Not reported	≥ 5 prescription medications
Farrell et al. (2020) Canada [64]	The ‘study sought to gain an in‐depth understanding of how prescribing cascades develop and persist, and to identify potential strategies … to … identify, prevent or manage prescribing cascades’	Design—Exploratory descriptive Method—Qualitative study using semi‐structured interviews Framework—Not reported	Instrument—Author‐designed interview guide Validation—Not reported	Primary care	*n* = 8 patients *n* = 1 caregiver *n* = 5 HCPs	Age 70–95 years Male gender—50%	Of the 5 HCPs, *n* = 1 pharmacist, rest physicians	> 1 prescription medication
Fried et al. (2017) The United States [39]^b^	‘To examine the effects of Tool to Reduce Inappropriate Medications (TRIM) on shared decision‐making about medications’	Design—Randomised clinical trial Method—Quantitative study using audio recordings of clinic visits followed by interviews and chart reviews	Instrument—Adapted Patient Assessment of Chronic Illness Care Validation—Not reported Instrument—Active Patient Participation Coding Scheme Validation—Previously validated Counting utterances pertaining to medications	Primary care	*n* = 64 intervention (*n* = 55 recordings) *n* = 64 control Response rate 34%	Age 65–69 years *n* = 52 (40.6%) Age 70–79 years *n* = 57 (44.5%) Age ≥ 80 years *n* = 19 (14.8%) Male gender—98.4%	75.8% Caucasian 51.6% married 32% of participants had some tertiary education or higher 68.5% of participants reported they either had just enough money left over each month or not enough 19.5% employed	≥ 7 regular medications with one of these for either hypertension or diabetes
Gillespie et al. (2019) Australia [40]^a^	The ‘study aimed to explore attitudes, beliefs and experiences regarding polypharmacy and deprescribing among community‐living older adults, taking five or more medications’	Design—Exploratory Method—Quantitative study using a self‐administered survey	Instrument—Adapted 3 items—Canadian Survey of Experiences with Primary Health Care Validation—Not reported	Community	*n* = 137 Response rate 25.1%	Age 76 (IQR: 73–83) Male gender—38%	93.4% born in Australia, New Zealand or the United Kingdom 50.4% of participants were of low socio‐economic status 51.1% received a Trade or University education	≥ 5 prescription medications
Gillespie et al. (2022) Australia [42]^a^	The ‘study aimed to explore the factors which influence GP and older adult decisions about deprescribing in primary care … to understand why prescriber and older adult willingness to deprescribe do not translate into action’	Design—Exploratory Method—Qualitative study using semi‐structured interviews Framework—Not reported	Instrument—Author‐designed interview guides Validation—Pilot tested	Community	*n* = 25 older people *n* = 16 GPs	Age 79 (67–95) Male gender—44%	92% born in Australia or the United Kingdom 80% reported good health status and quality of life 56% lived in a low socio‐economic area	≥ 5 prescription medications
Gillespie et al. (2023) Australia [41]^a^	This study ‘measured older adult health literacy…in the management of polypharmacy and in decisions about deprescribing’ and ‘investigated GPs' descriptions of their interactions with their older patients regarding medication decision‐making’	Design—Sequential explanatory Method—Mixed Quantitative study using surveys followed by Qualitative semi‐structured interviews Framework—Not reported	Instrument—Author‐designed GP survey Validation—Pilot tested for usability and face validity Instrument—Adapted 3 items—Canadian Survey of Experiences with Primary Health Care Validation—Not reported Instrument—Author‐designed interview guides Validation—Not reported	Community	Phase 1^a^: *n* = 137 older adults *n* = 85 GPs Phase 2^a^: *n* = 25 older people *n* = 16 GPs	Phase 1^a^: Age 76 (IQR: 73–3) Male gender—38% Male gender (GP)—57% Phase 2^a^: Age 79 (67–95) Male gender—44% Male gender (GP)—62.5%	See Gillespie (2019; 2022)^a^	≥ 5 prescription medications
Green et al. (2020) The United States [87]	The study examined ‘how primary care clinicians discuss medications during encounters with older adults with cognitive impairment and their companions’	Design—Content analysis Method—Qualitative study using audio recordings of clinic visits Framework—Not reported	Instrument—Audio‐recorded clinic visits Instrument— Deductive coding guided by SDM framework by Elwyn et al. (2023)	Primary care	*n* = 93 patients *n* = 93 companions *n* = 14 HCPs	Age 79.9 (7.6) Male gender (patient)—48.4% Male gender (companion)—24.7% Male gender (HCP)—50%	Of the HCPs, *n* = 5 nurse practitioner/doctor assistant, rest doctors Of the companions, 39.8% spouse/partner; 54.8% adult child. 48.4% of patients had greater than high school education.	Cognitive impairment
Haverhals et al. (2011) The United States [65]	To ‘understand the medication self‐management needs and strategies of older adults and their adult caregivers that could be addressed through effective personal health application design’	Design—Exploratory Method—Qualitative study using focus groups followed by semi‐structured interviews Framework—Not reported	Instrument—Author‐designed interview guides Validation—Pilot tested	Community	*n* = 32 older adults *n* = 2 caregivers	Age 82 (73–90)ª Male gender (older adult)—40%ª Male gender (caregiver)—0%ª	82.4% Caucasianª	≥ 1 chronic health conditions ≥ 3 regular prescription medications
Højgaard et al. (2024) Denmark [78]	To explore participants' ‘perspectives on and decisions regarding initiating cardiovascular preventive medication for screen‐detected cardiovascular disease’	Design—Exploratory Method—Qualitative study using semi‐structured interviews Framework—Paterson's Model of Chronic Illness	Instrument—Author‐designed interview guides Validation—Pilot tested	Community	*n* = 12	Age 68 years Male gender—58.3%	66.7% married; 33.3% living alone	Diagnosed with carotid plaque Recommended to take preventive medication for cardiovascular disease
Holmqvist et al. (2019) Sweden [59]	The ‘study explored and described older persons' experiences of evaluation of their medication treatment’	Design—Exploratory descriptive Method—Qualitative study using semi‐structured interviews Framework—Medication Use Model	Instrument—Author‐designed interview guides Validation—Pilot tested	Community	*n* = 20	Age 81 (75–91) Male gender—55%	Not reported	≥ 1 chronic health conditions ≥ 1 regular prescription medication
Hu et al. (2023) China [55]	The study aimed ‘to find the factors associated with participation of patients with chronic diseases in shared decision‐making on medication’	Design—Correlational Method—Quantitative using face‐to‐face questionnaires	Instrument—Control Preference Scale‐Post Validation—Tool previously validated	Community	*n* = 901^d^ Response rate = 99.3%	Age 65–75 years *n* = 641 (71.1%)^d^ Age ≥ 75 years *n* = 260 (28.9%)^d^ Male gender—42.06% (overall study)	All living in rural or urban China	Hypertension or diabetes ≥ 1 regular prescription medication
Jansen et al. (2019) Australia [53]	To ‘explore older people's perspectives and experiences with decisions about medication for CVD prevention’	Design—Exploratory descriptive Method—Qualitative study using semi‐structured interviews Framework—Not reported	Instrument—Author‐designed interview guides Validation—Pilot tested	Community	*n* = 30	Age > 75 years (75–90+) Male gender—53.3%	80% born in Australia; 66.7% married; 20% widowed; rest single/divorced 73.3% had a high school education or less	Diabetes with elevated risk factors for CVD ≥ 1 regular prescription medication
Junius‐Walker et al. (2021) Germany [63]	To test if the electronic communication tool MediQuit will promote SDM and reduce polypharmacy	Design—Descriptive Method—Quantitative using an interviewer‐administered telephone survey (patients); self‐administered questionnaire (GPs)	Instrument—Author‐designed surveys Validation—Not reported	Community	*n* = 37 interviewed *n* = 4 lost to follow‐up	Age 77.1 (8.1) Male gender—39%	Not reported	≥ 3 chronic conditions ≥ 5 prescription medications
Kempen et al. (2020) Sweden [80]	To ‘explore older patients' experiences with and views on hospital initiated comprehensive medication reviews and follow‐up telephone calls by ward‐based clinical pharmacists’	Design—Exploratory descriptive Method—Qualitative study using semi‐structured interviews Framework—Interpretive approach	Instrument—Author‐designed interview guide Validation—Not reported	Hospital inpatient	*n* = 15 (3 included spouse/carer) Response rate = 41.6%	Age 66–94 Male gender—53%	Not reported	≥ 1 regular prescription medication
Knight et al. (2013) The United Kingdom [79]	‘To explore older people and their carers' experience of hospital discharge regarding patient‐centred care, shared decision making and the organization and management of medicines’	Design—Exploratory descriptive Method—Qualitative study using semi‐structured interviews Framework—Not reported	Instrument—Author‐designed interview guide Validation—Not reported	Community	*n* = 7 older people *n* = 12 carers	Older person and care recipient: Age 84 (75–100) Male gender—26.3%	Not reported	Recently discharged from hospital ≥ 4 regular prescription medications
Kreling et al. (2006) The United States [66]	Understanding the factors for the use of adjuvant non‐hormonal chemotherapy in older women	Design—Exploratory Method—Qualitative study using focus groups Framework—Not reported	Instrument—Author‐designed interview guide Validation—ongoing refinement	Community	*n* = 34	Age ≥ 65 years Male gender—0%	53% Caucasian, 29.4% African American, 17.6% Latino	Breast cancer
Lansbury (2000) Australia [62]	The ‘study explored the preferred strategies of elderly people and the barriers they encountered in trying to manage their pain’	Design—Exploratory Method—Qualitative study using focus groups followed by semi‐structured interviews Framework—Grounded theory	Instrument—Author‐designed interview guide Validation—Not reported	Community	*n* = 72	Age 75 (65–90) Male gender—19.4%	*n* = 1 upper middle class, *n* = 3 middle class and *n* = 2 working‐class suburbs 62.5% widowed	Chronic pain
Manias et al. (2024) Australia [50]	This study aimed to ‘explore decision‐making between health professionals, older patients and families about medication changes across transitions of care’	Design—Ethnography Method—Qualitative study using observations, semi‐structured interviews and reflexive focus groups Framework—Patient and Family engagement framework	Instrument—Author‐designed interview guides Validation—Pilot tested	Hospital inpatient	*n* = 182 patients *n* = 44 family members *n* = 94 HCPs	Patients: Age range 65–104 Male gender (patient)—50% Male gender (family member)—34.1%	Family members were partners/spouses (52.3%), son/daughter/son‐ or daughter‐in‐law (45.5%), and grandchild (2.2%) Of the HCPs, *n* = 12 were doctors; *n* = 12 were pharmacists and *n* = 70 were nurses	≥ 1 regular prescription medication
McCabe et al. (2019) The United Kingdom [28]	To ‘measure shared decision making when starting cholinesterase inhibitors’	Design—Exploratory Method—Qualitative study using video recordings of clinic visits followed by a self‐administered questionnaire Framework—Not reported	Instrument—Observing Patient Involvement in Decision Making scale; Patient Experience Questionnaire; Satisfaction with Decision Scale; Autonomy Preference Index Validation—Tools previously validated All tools assessed for internal consistency using Cronbach's *α*	Primary care	*n* = 74 patients *n* = 69 companions *n* = 21 doctors	Patients: Age 81.7 (65–91) Male gender (patient)—39.2% Male gender (companion)—43.5%	Of the patients, 89% Caucasian 55.4% married; 24.3% widowed; rest single/divorced 60.5% had a high school education or less Companions were mostly partners/spouses (43.5%) or family members (42%)	Dementia
Mc Gillicuddy et al. (2019) Ireland [67]	To ‘investigate the knowledge, attitudes and beliefs of community‐dwelling older adults and carers of community‐dwelling older adults about the modification of oral medicines’	Design—Exploratory descriptive Method—Qualitative study using semi‐structured interviews Framework—Not reported	Instrument—Author‐designed interview guides Validation—Pilot tested	Community	*n* = 13 older people *n* = 13 caregivers	Age 77 (IQR 72.5–84) Male gender (older person)—38.5% Male gender (caregiver)—7.7%	Of the carers, 69.3% were related to the recipient, and the rest were paid as carers	≥ 1 regular prescription medication that requires to be modified or has difficulty swallowing
Mecca et al. (2022) The United States [43]^b^	‘To perform a mixed‐methods evaluation of the clinical interactions between patients and clinicians in the TRIM study to examine in greater detail the communication and decision making around medications and deprescribing’	Design—Descriptive/Inductive Method—Mixed Quantitative descriptive analysis and Qualitative inductive analysis of audio transcripts Framework—Not reported	Instrument—Texts labelled according to TRIM report, recommendation for change in medication and patient involvement	Primary care	*n* = 113 of the original 119 recordings available	Same as Fried et al. (2017)^b^	Same as Fried et al. (2017)^b^	Same as Fried et al. (2017)^b^
O'Quinn et al. (2015) The United States [82]	To ‘ascertain caregiver and elder perceptions of barriers to medication management and to identify community‐derived solutions to improve medication management’	Design—Exploratory descriptive Method—Qualitative study using focus groups Framework—Not reported	Instrument—Author‐designed interview guide Validation—Stakeholder input	Community	*n* = 48 older people *n* = 17 caregivers Response rate ‐ Not reported	Age 72 (mean) Male gender (older person)—27% Male gender (caregiver)—12%	76% and 100% Caucasian 67%% and 69% had greater than high school education 11% of older people employed	≥ 1 regular prescription medication
Ouellet et al. (2022) Canada [72]	To understand ‘how prescribing is perceived by very old adults, caregivers and health professionals’	Design—Exploratory descriptive Method—Qualitative study using focus groups and semi‐structured interviews Framework—Not reported	Instrument—Author‐designed interview guides Validation—Not reported	Community	*n* = 10 older people *n* = 6 caregivers *n* = 11 HCPs	Age 80–93 (range) Male gender (older person)—10% Male gender (caregiver)—66.6% Male gender (HCP)—63.6%	Of the HCPs, *n* = 4 were doctors; *n* = 2 were pharmacists and *n* = 5 were nurses	Average 9.4 medications per day
Parekh et al. (2019) The United Kingdom [81]	The ‘study explored the lived experience of medication‐related problems in older adults with varying functional levels, focussing on the hospital discharge period’	Design—Exploratory descriptive Method—Qualitative study using focus groups and semi‐structured interviews Framework—Not reported	Instrument—Author‐designed interview guides Validation—Stakeholder input	Community	*n* = 18 older people *n* = 2 caregivers	Age 78 (65–98) Male gender—30%	All Caucasian	Experience of a recent medication‐related problem
Peat et al. (2023) The United Kingdom [57]	The study aimed to ‘present deprescribing experiences of patients living with frailty, their informal carers and healthcare professionals’	Design—Exploratory descriptive Method—Qualitative study using semi‐structured interviews Framework—Not reported	Instrument—Author‐designed interview guides Validation—Stakeholder input	Primary care	*n* = 9 older people *n* = 4 caregivers *n* = 14 HCPs	Older person: Age > 70 years Male gender (older person)—66.7% Male gender (caregiver)—0% Male gender (HCP)—7.1%	All Caucasian Of the HCPs, *n* = 6 were doctors; *n* = 3 were pharmacists/technicians and *n* = 5 were advanced practice nurses	Had a medication deprescribed
Perreira, Bieri, del Rio Carral et al. (2022) Switzerland [45]^c^	The ‘study aimed to describe the perceived needs for collaborative medication management for older adults taking several different medications at home after hospital discharge’	Design—Descriptive Method—Qualitative study using semi‐structured interviews Framework—Not reported	Instrument—Author‐designed interview guides Validation—Pilot tested with ongoing refinement	Community	*n* = 28 older people *n* = 17 caregivers *n* = 13 HCP	Age 81 (66–94) Male gender—60.7%	Of the HCPs, *n* = 4 were doctors; *n* = 4 were pharmacists and *n* = 5 were nurses	≥ 3 chronic conditions ≥ 5 prescription medications
Perreira, Bieri, Martins et al. (2022) Switzerland [44]^c^	The ‘study aimed to identify and categorise the stressors experienced and reconstitution strategies adopted by older adults, their informal caregivers, and healthcare professionals as they manage older adults' medications after hospital discharge’	Design—Exploratory descriptive Method—Qualitative study using semi‐structured interviews Framework—Neumann Systems Model	Instrument—Author‐designed interview guides Validation—Pilot tested	Community	*n* = 28 older people *n* = 17 caregivers *n* = 13 HCP	Age 81 (66–94) Male gender—60.7%	Of the HCPs, *n* = 4 were doctors; *n* = 4 were pharmacists and *n* = 5 were nurses	≥ 3 chronic conditions ≥ 5 prescription medications
Reeve et al. (2016) Australia [73]	‘To explore the views, beliefs, and attitudes of older adults and carers on deprescribing’	Design—Exploratory Method—Qualitative study using focus groups Framework—Not reported	Instrument—Author‐designed interview guide Validation—Not reported	Community	*n* = 14 older people *n* = 14 carers	Age ≥ 65 years Male gender—57%	93% of carers were family members	≥ 1 chronic health condition ≥ 1 regular prescription medication
Ross and Gillett (2021) Canada [74]	The study was ‘to understand how older adults make healthcare decisions through axes of trust that operate across the system‐world and lifeworld’	Design—Exploratory Method—Qualitative study using semi‐structured interviews Framework—Communicative Action (Habermas)	Instrument—Not reported Validation—Not reported	Primary care	*n* = 16	Age 81 (73–90) Male gender—31%	Not reported	Not reported
Sale et al. (2011) Canada [75]	To ‘examine patients' experiences with the decision to take osteoporosis medication after they sustained a fracture’	Design—Eidetic phenomenology Method—Qualitative study using semi‐structured interviews Framework—Not reported	Instrument—Author‐designed interview guide Validation—Not reported	Community	*n* = 21 Response rate = 70%	Age 65–88 Male gender—28.6%	Not reported	Deemed high risk for fractures Prescribed medication for osteoporosis prevention
Salter et al. (2014) The United Kingdom [83]	To ‘explore the factors that influence older women's adherence to prescribed prophylactic medication when assessed to have higher than average risk of fracture’	Design—Exploratory longitudinal Method—Qualitative study using semi‐structured interviews Framework—Not reported	Instrument—Author‐designed interview guide Validation—Not reported	Community	*n* = 30	Age 73–85 Male gender—0%	Not reported	Deemed high risk for fractures Prescribed medication for osteoporosis prevention
Schmittdiel et al. (2010) The United States [85]	To ‘assess Medicare Part D beneficiaries with diabetes' levels of communication with physicians regarding prescription drug costs’	Design—Exploratory Method—Quantitative cross‐sectional survey	Instrument—Author‐designed survey Validation—Not reported	Community	*n* = 1458 response rate = 58%	Age 75 (5.8) Male gender—45.6%	74% Caucasian; 16% Latino 79% of participants had a high school/some college education or less	Diabetes
Schopf et al. (2018) Germany [68]	To ‘explore elderly patients' and GPs' perceptions of communication about polypharmacy, medication safety and approaches for empowerment’	Design—Exploratory Method—Qualitative study using semi‐structured interviews Framework—Not reported	Instrument—Author‐designed interview guides Validation—Not reported	Primary care	*n* = 6 patients *n* = 3 HCPs	Age 75 (4.8) Male gender (patient)—50% Male gender (HCP)—100%	66.7% of patients had a high school education or less Of the HCPs, *n* = 1 GP, *n* = 1 GP assistant and *n* = 1 final year medical student	≥ 5 regular prescription medications
Smith et al. (1994) The United States [60]	To explore the perceptions of the older elderly about communication about their medications	Design—Retrospective Method—Quantitative using a self‐administered questionnaire	Instrument—Author‐designed questionnaire Validation—Not reported	Community	*n* = 110 over the counter *n* = 218 prescription Response rate 73%	Over the counter: Age 77 (mean) Prescription: Age 83 (mean) Overall Age 65–93 Male gender (OTC)—31% Male gender (prescription)—37%	> 50% living with their spouse About 40% widowed Education: 13 years (mean)	Not reported
Spinewine et al. (2005) Belgium [76]	‘To explore the processes leading to inappropriate use of medicines for elderly patients admitted for acute care’	Design—Exploratory Method—Qualitative using observations of ward practices, focus groups and semi‐structured interviews Framework—Grounded theory	Instrument—Author‐designed interview guides Validation—Pilot tested	Hospital inpatient	*n* = 17 patients *n* = 12 HCPs	Patients: Age 73–92 years Male gender (patient)—41.2% Male gender (HCP)—33.3%	Of the HCPs, *n* = 5 doctors, *n* = 4 nurses, *n* = 3 pharmacists	≥ 1 chronic health condition ≥ 1 regular prescription medication and requiring at least one medication change
Thevelin et al. (2022) Europe [49]	‘To explore experiences of hospital‐initiated medication changes in older people with multimorbidity’	Design—Exploratory descriptive Method—Mixed Qualitative using semi‐structured interviews and Quantitative using self‐administered surveys Framework—National Health Service Patient Experience Framework	Instrument—Author‐designed interview guide Validation—Pilot tested Instrument—Shared decision‐making questionnaire—physician version Validation—Tool previously validated	Community	*n* = 48 patients *n* = 17 HCPs	Age ≥ 70 years Male gender—52%	Of the patients: *n* = 15 Belgium; *n* = 7 Ireland; *n* = 11 Switzerland; *n* = 15 the Netherlands 62.5% of patients had a high school education or less	≥ 3 chronic health conditions ≥ 5 regular prescription medications
Tietbohl & Bergen (2022) The United States [77]	To ‘describe how patients' question design can highlight their engagement in medical care’	Design—Descriptive Method—Qualitative using video recordings of clinic visits Framework—Not reported	Instrument—Conversation analysis: agency framing and Jeffersonian transcription	Primary care	*n* = 52	Age 81 (68–98) Male gender—34%	66% Caucasian	Not reported
Tinetti et al. (2024) The United States [61]	The study aimed ‘to evaluate the association between receiving patient priorities care or usual care and patient‐reported outcomes and days not at home because of health’	Design—Non‐randomised clinical trial Method—Quantitative using an interviewer‐administered questionnaire	Instrument—CollaboRATE Validation—Tool previously validated Instrument—Accountable Care Organisation shared prescribing decision‐making quality measure Validation—Not reported	Primary care	*n* = 129 intervention *n* = 135 control	Intervention: Age 75.6 (6.5) Control: Age 75.3 (6.1) Male gender (intervention)—57.4% Male gender (control)—51.1%	90.5% Caucasian 26.5% of the participants had a high school education or less 35.6% of the participants lived alone	≥ 3 chronic health conditions Taking multiple medications
Tinetti et al. (2019) The United States [47]	‘To evaluate whether patient priorities care is associated with a perception of more goal‐directed and less burdensome care compared with usual care’	Design—Non‐randomised clinical trial Method—Quantitative using an interviewer‐administered questionnaire	Instrument—Older Patient Assessment of Chronic Illness Care; CollaboRATE Validation—Tools previously validated	Primary care	*n* = 163 intervention *n* = 203 control	Intervention: Age 77.6 (7.6) Control: Age 74.7 (6.6) Male gender (intervention)—33.1% Male gender (control)—37.9%	95.6% Caucasian 53.4% of the participants had a high school education or less	≥ 3 chronic health conditions Taking multiple medications
Tjia et al. (2008) The United States [69]	The study aimed ‘to explore the concerns of older adults with diabetes about the complexity of their drug regimens and to determine whether they discussed medication‐related concerns with their physician’	Design—Exploratory descriptive Method—Qualitative study using semi‐structured interviews Framework—Not reported	Instrument—Author‐designed interview guide Validation—Not reported	Primary care	*n* = 22	Age 75 (7.2) Male gender—27.2%	73% African American 41% married; 27% widowed; 27% divorced 77% of participants had a high school/some college education or less	Type 2 diabetes ≥ 5 regular prescription medications
Tobiano et al. (2021) Australia [27]	The ‘study aimed to examine older medical patient and family participation in discharge medication communication’	Design—Explanatory Method—Mixed Quantitative (using observation of discharge medication communication and self‐administered questionnaire) to Qualitative study (using semi‐structured telephone interviews) Framework—Not reported	Instrument—Author‐designed observation tool Validation—Content validity undertaken Instrument—Author‐designed interview guides Validation—Not reported	Hospital inpatient	Phase 1 *n* = 30 Phase 2 *n* = 8 *n* = 3 family members	Age 76.8 (7.2) Male gender—53.3%	83.7% of participants had a high school education or less	≥ 1 chronic health condition ≥ 6 regular prescription medications
Weir et al. (2021) Australia [54]	The study ‘explored GPs' perspectives on the importance of discussing patients' goals and preferences, and the role patient preferences play in medicines management and prioritisation’	Design—Phenomenology Method—Qualitative study using semi‐structured interviews Framework—Not reported	Instrument—Author‐designed interview guide Validation—Pilot tested	Primary care	*n* = 32 GPs	Age not reported Male gender—43.7%	31.3% of GP practices had up to 19% of their patients over 75 years, and 21.9% of practices had > 40% of their patients aged 75 years and older 50% of GPs practised in the least disadvantaged socio‐economic areas, with 31.3% practising in the most disadvantaged areas	
Weir et al. (2018) Australia [46]	The ‘study explores decision‐making about polypharmacy with older adults and their companions’	Design—Phenomenology Method—Qualitative study using semi‐structured interviews Framework—Not reported	Instrument—Author‐designed interview guide Validation—Not reported Instrument—Control preference scale (CPS) Validation—Tool previously validated	Community primary care	*n* = 30	Age ≥ 75 years Male gender—36.7%	*n* = 15 partners and *n* = 4 other companions 66.7% of participants had a high school education or less	Taking multiple medications
Wilson et al. (2007) The United States [84]	‘To determine the prevalence of physician–patient dialogue about medication cost and medication adherence among elderly adults’	Design—Cross‐sectional Method—Quantitative using a self‐administered survey	Instrument—Author‐designed survey Validation—Not reported	Community	*n* = 17,569 (*n* = 15,445 taking medication) Response rate 51%	Age 74.9 (7) Male gender—41.3%	88% Caucasian 74.8% had greater than higher school education 50.7% of older people had a > 5‐year relationship with their HCP 57.2% of low socio‐economic status	≥ 1 regular prescription medication
Xu et al. (2003) The United States [48]	The ‘study examined patient–provider communication regarding drug affordability from a consumer's perspective’	Design—Cross‐sectional Method—Quantitative using an interviewer‐administered survey	Instrument—Participatory decision‐making (PDM) Validation—Tool previously validated Instrument—Author‐designed question Validation—Not reported	Community	*n* = 2360 Response rate 45.2%	Age > 65 years Age 65–70 *n* = 954 (40.42%) Age 71–75 *n* = 645 (27.33%) Age 76–80 *n* = 451 (19.11%) Age ≥ 81 *n* = 310 (13.14%) Male gender—31.9%	83.8% Caucasian; 12% Hispanic 57% of participants had less than a high school education 44.3% lived in semi‐rural or rural areas > 54% of low socio‐economic status	≥ 1 regular prescription medication

Abbreviations: CVD, cardiovascular disease; GP, general practitioner; HCP, healthcare provider; SDM, shared decision‐making.

a Articles reporting the same study.

b Articles reporting the same study.

c Articles reporting the same study.

d Only data from participants aged ≥ 65 years were extracted.

### 3.3 Study Findings

Findings of this review related to the decision‐making practices of the HCP and the patient and/or carer when medication changes were made, or a new medication was prescribed, and were organised into three themes: descriptions of *provider‐driven decision‐making, patient‐driven decision‐making and a shared role in decision‐making*.

#### 3.3.1 Provider‐Driven Decision‐Making

Provider‐driven DM was identified in 38 studies (Appendix C). Defining features of this style were that the HCP made the decision about a medication without input from the patient, and the patient then followed the decision without question. Within the community and primary care setting, several participants indicated they were not involved in the decision to prescribe a medication or were not offered further information about their medication [40, 46, 51, 57, 59, 62]. In five studies, between 15.6% and 77% of patient and doctor participants reported provider‐driven DM [40, 46, 49, 55, 63]. Whilst most of the studies included small sample sizes (*n* = 30–137), mostly very old people (aged ≥ 75 years) with multiple chronic conditions and those taking more than five medications, another common feature was the high percentage of participants with less than high school education. According to Gillespie et al. [41], in their survey of doctors, 49.4% (*n* = 42) thought older patients would prefer provider‐driven DM. Tobiano et al. [27] undertook observations of communication episodes between patients and HCPs related to medication management in the hospital inpatient setting. The patient was considered not involved in 29.6% (*n* = 21) of 71 medication‐related encounters [27]. Using the data collection tool OPTION‐5 to assess video recordings of clinic consultations with older people diagnosed with dementia, McCabe et al. [28] reported less patient involvement occurred when more than three people were present (e.g., additional HCPs) during the consultation and that the lowest OPTION score (Mean 0.34, SD 0.69) related to supporting ‘the patient in becoming informed’ and deliberating about the options. In nine qualitative studies, patients preferred provider‐driven DM; however, they did not clarify why this approach was preferred [53, 54, 57, 64, 65, 66, 67, 68, 69], while in other studies, the main reason for provider‐driven DM preference was found to be patient trust and confidence in the HCP's expertise [15, 41, 46, 49, 50, 56, 58, 59, 70, 71, 72, 73, 74, 75, 76, 77, 78].

Many participants experienced medication changes during their hospital inpatient stay without being informed or involved in the DM process [44, 45, 49, 50, 67, 76, 79, 80, 81]. Reasons for this were reported to be related to the post‐surgical period or declining health [49, 64, 80]. However, according to Knight et al. [79], the patient and/or their carer were often unaware of medication changes even at discharge, finding out about changes when they returned home. Patients' and carers' perceived inability to engage in SDM was reported in 20 studies.

Reasons included fear, anxiety and feeling overwhelmed by their illness [15, 64, 66], especially in situations of increasing morbidity [64, 71]. Many patients and carers desired involvement in SDM; however, they lacked confidence in their abilities to ask due to a lack of knowledge about the medication or disease process [15, 41, 42, 44, 45, 46, 49, 58, 72, 74, 82]. In four studies, HCPs' interpersonal skills were reported as reasons for patients and carers being unable to participate in the SDM process [15, 62, 71, 82]. Interpersonal skills of HCPs that discouraged a collaborative approach included using medical terminology that patients could not understand, inadequate information, insufficient opportunity for questions, not listening to patient concerns or deflecting the conversation [15, 45, 59, 60, 62, 70, 82].

According to Belcher et al. [15], the reaction of the HCP could lead to a more collaborative interaction or not. Analysis revealed that some older people needed encouragement to participate in SDM and that unless asked, patients would often not speak up [15, 52, 68]. From the HCP perspective, a perceived lack of time, concern about poor health literacy levels or attitudes towards ageing and perceived frailty were identified as reasons for not involving the older person in SDM [41, 42, 52].

#### 3.3.2 Patient‐Driven Decision‐Making

Analysis revealed few participants preferred patient‐driven DM (Appendix C) [46, 53, 55, 63, 65, 67, 69, 75, 78, 80, 83]. The defining features of this style were that the patient was prescribed a medication and then decided whether to take the medication with or without input from their HCP. Two of the 71 communication encounters in the study by Tobiano et al. were described as autonomous DM [27]. In a quantitative study by Hu et al. 5.3% (*n* = 48) of patients using the Control Preference Scale‐Post (CPS‐Post) reported patient‐led DM [55]. According to Gillespie et al. [41] and Junius‐Walker et al. [63], who surveyed doctors, 8% (*n* = 3) and 12.9% (*n* = 11) of doctors, respectively, thought older patients would prefer patient‐driven DM. There were reports of six patients initiating discussions with their HCP about medication and then making their own decision to take or alter the dose of that medication [49, 53, 70, 78]. For two patients, this related to adverse effects from their cardiovascular medications, for which the HCP offered a recommendation; the patient was then left to decide whether to cease or alter the dose of the medication [53, 70]. Two patients were recommended for and subsequently refused preventative medicines following cardiovascular disease screening [78]. The remaining two patients described situations where, as an inpatient, they made the decision to either commence a new medication or not, regardless of the HCP's recommendations [49].

According to Sale et al. [75] and Tjia et al. [69], many patient‐driven decisions were made following a clinic visit, and according to some participants, this was the result of poor communication [65, 75, 79]. In these instances, patients would decide not to have their medication prescription filled at the pharmacy after seeing their HCP. Qualitative analysis identified that participants who decided to either stop or change the dose of medication themselves would then either tell their HCP afterwards or not tell them at all [46, 51, 65, 67, 68, 79, 80, 83, 84]. The main reasons for changing a medication regimen were related to side effects, the perception of a medication's ineffectiveness and medication cost. Decisions about nonadherence were most often made by patients at risk of fractures or cardiovascular disease, or prescribed increasing numbers of medications.

#### 3.3.3 Shared Role in Decision‐Making

Patients engaging in discussions with their HCP about medications or having an active role in medication‐related DM were reported in most studies (Appendix C). The defining features of this style were the patient's and HCP's active role in discussing medications so that collaborative decisions could be made. SDM mostly included decisions between the HCP and patient only; however, at times, others (e.g., carer, another HCP, such as another doctor, pharmacist or nurse) participated in the SDM process as part of the team. There were a number of defining features of the research findings in relation to the SDM role, which are reported in the following sections, including the topics of discussion, personal responsibility, asking questions, use of interventional tools and conceptual understanding.

##### 3.3.3.1 Topics of Discussion in the SDM Process

In their mixed methods study, Tobiano et al. [27] used clinical observations to identify SDM in 33.8% (*n* = 24) of medication‐related interactions. These interactions were with a pharmacist (*n* = 21) or a nurse (*n* = 3); however, 16 of the shared decisions related to the patient's preferred pharmacy for medication access [27]. In another mixed‐methods study, Gillespie et al. [40, 41] included an adapted primary healthcare survey asking patient participants if their HCP had involved them in medication‐related decisions, with 30% (*n* = 41) indicating ‘sometimes’ and 41.6% (*n* = 57) reporting ‘often’ [40]. In the study by Hu et al. 35.4% (*n* = 319) reported SDM when prescribed a new medication or having a medication change [55]. The data from both studies showed a positive correlation between higher health literacy and older people actively participating in SDM [41, 55].

Patients and carers described how they contributed to decisions about medication preferences and goals of care. Discussions on patient preferences included topics such as the medication's purpose, alternative medication options, impact on functioning and the ability to alleviate symptoms [28, 71, 75, 78, 80, 83]. Collaborative discussions also covered topics related to side effects [42, 46, 50, 53, 56, 57, 58, 64, 84] or other medication‐related issues (e.g., cost and administration challenges) [27, 48, 67, 84, 85].

##### 3.3.3.2 Responsibility for Involvement in SDM

Findings highlighted how participants assumed personal responsibility for involvement in SDM. Responsibility, in the findings, appeared to refer to the persons' duty and ownership of the task to be involved in discussions related to SDM. The findings highlighted a shift in the onus of responsibility over time, with the earlier papers reporting HCP encouragement for patient involvement in decision‐making [51, 60], and in the last decade, increasingly older adults are reporting their own ability to lead discussions through asking questions [27, 39, 49, 58, 64, 67, 77, 86].

Some patients and carers initiated discussions with their HCP to reduce the number of medications they were taking [15, 57, 58, 65, 72, 82, 83]. Some patients and carers sought information through reading package inserts, accessing the internet and then actively asking questions of HCPs [15, 41, 42, 77].

From the HCP perspective, Weir et al. [54] described how some HCPs preferred patients who got involved and asked questions, and the HCPs routinely encouraged this practice. Gillespie et al. [41] surveyed HCPs about older patients' capabilities to engage in SDM. Nearly 85% (*n* = 72) of HCPs believed that older people could engage in DM about their medications, and 63.5% (*n* = 54) thought older patients would prefer a shared role in DM. Similar to the findings of Weir et al. [54], HCPs in the study by Gillespie et al. [41, 42] encouraged patient involvement in medication discussions by asking questions about preferences. This encouragement was also evident in other studies where some HCPs tried to engage older persons, including those with cognitive impairment, in SDM discussions about medications [43, 48, 52, 69, 87, 88].

##### 3.3.3.3 Asking Questions to Promote Involvement in Decision‐Making

Asking questions to promote involvement in discussions and DM about was identified in 22 studies. Five papers referred to the HCP asking or not asking questions [40, 59, 60, 68, 69]; however, asking questions about medications was identified in 10 studies to be an important skill for patients [15, 39, 51, 54, 60, 64, 68, 77, 79, 80]. Preparation for the health visit by bringing a list of medications and questions was deemed essential for a collaborative environment [15, 39, 77]. Reasons for not asking questions included patient concern that the question might seem ‘silly’ [81], trust in the HCP [41, 42, 72] and the patient forgetting to ask or not wanting to bother the HCP [62, 67, 74]. Older people and carers actively asking questions or being encouraged to ask questions promoted involvement in SDM and self‐efficacy in medication management [27, 52, 53, 59, 60, 75, 77].

##### 3.3.3.4 Interventional Tools to Support SDM

In four studies, interventions to promote medication‐related discussions and SDM were tested [39, 47, 61, 86]. In two non‐randomised clinical trials (using the same intervention), one following the other, perceived engagement in the DM process was considered high at baseline for both the control and intervention groups, and the researchers reported that the intervention of identifying patient priorities before the clinic appointment did not impact DM [47, 61]. Following the second study, the authors suggested this may be a result of patients identifying the CollaboRATE questions more closely linked to patient satisfaction than to SDM [61]. Fried et al. [39] conducted a randomised clinical trial, providing an individualised patient feedback report on medications to both the patient and HCP. The intervention was found to increase patient engagement and HCP facilitation. Secondary analysis of the consultation audio recordings was carried out by Mecca et al. [43], who extended upon the results of Fried et al.'s study, stating that HCPs attempted to engage patients in SDM using the interventional tool in 13.2% (*n* = 7) of the intervention participants. Campbell et al. [86] explored the efficacy of an educational and support programme, evaluating the benefits of a customised letter provided to patients for their HCPs. Seven of the 15 patients who presented the letter had subsequent discussions, leading to increased patient engagement and medication‐related DM [86].

##### 3.3.3.5 Understanding of SDM

Qualitative research highlights variations in participant understanding of SDM related to medications. According to Belcher et al. [15], participants in pilot study interviews lacked an understanding of the term ‘shared decision‐making’; therefore, the authors re‐phrased their interview questions to promote participant understanding of the concept. Jansen et al. [53] highlighted that many older people (aged ≥ 75 years) thought medications were a necessity and did not recognise that medication prescribing was a decision to be considered. These participants either had experienced a cardiovascular event, had a family history of chronic conditions or had limited knowledge of risk versus actual disease. Further, a high percentage (73.3%, *n* = 22) of these participants had a high school education or less [53]. Medication‐related SDM was described by some patients and carers as just being provided with information about medications (including the purpose, how to take the medication, side effects and/or changes to medication regimens), being more involved in information exchange and discussion about medication options, and being involved in actual DM [46, 53, 62, 64, 65, 71, 73]. Weir et al. [46] reported that 70% (*n* = 21/30) of participants preferred provider‐driven DM when surveyed using the CPS. However, it was revealed in the interviews that some participants were involved in discussions about their medications and left the final decision to their HCP with their preferences taken into consideration [46].

A discrepancy in the conceptual understanding of SDM between patients and HCPs was highlighted in two studies [49, 63]. Junius‐Walker et al. [63] undertook a pilot study to determine the feasibility of an SDM communication tool to promote deprescribing in older people. Following the consultation, HCPs were asked about patient involvement in the decision to deprescribe, with HCPs reporting that in 70% (*n* = 26) of consultations, SDM had occurred. However, when patients were asked the same question, only 49% (*n* = 18) reported that the decision had been shared. Of these 37 consultations, only 43% (*n* = 16) of the HCPs and patients agreed about the decision‐making approach [63]. Thevelin et al. [49] undertook a multi‐centre study to explore inpatient experiences of medication changes. In the total sample, 23% (*n* = 11/48) of patient participants reported experiencing SDM whilst in hospital. In a subset of participants (*n* = 27) from the intervention arm of the main study, a sample of HCPs (*n* = 17) using the validated SDM questionnaire for doctors (SDM‐Q‐Doc) perceived high levels of patient involvement in medication‐related DM with a median score of 76 out of 100 [49]. It was also reported that 70% (*n* = 19/27) of medication review consultations consisted of SDM. However, according to patient perceptions in the interviews, only 30% (*n* = 8/27) reported SDM.

##### 3.3.3.6 Involvement of Another HCP or Companion

Having another person present at medical appointments with the older person was seen to promote collaborative discussions and SDM about medications [27, 49]. This was usually a partner/spouse or family member. The advantages of having a companion present included knowing the recipient well [44, 49, 59, 65, 67, 73, 87], writing notes [66], asking questions [68, 82] and hearing things or reminding the patient of anything that may have been missed [66, 68].

Having the support of another HCP, often outside of the consultation, was also seen to promote collaborative discussions and DM about medications. Findings from four studies supported the involvement of other HCPs, such as pharmacists and nurses, who were able to identify medication‐specific issues (e.g., formulation issues for a patient with swallowing difficulties) and promote collaborative discussions and decisions that incorporated patient preferences [52, 67, 70, 76].

## 4 Discussion and Conclusion

### 4.1 Discussion

This systematic review examined the DM practices of older people, carers and HCPs related to medication management. Decision‐making about medication management between older people, carers and HCPs was provider‐driven, patient‐driven and shared. Provider‐driven DM was commonly reported in the hospital setting. In the community, older people who preferred provider‐driven DM did so because of trust in the HCP or as a result of increasing morbidity, anxiety or lack of knowledge. Patient‐driven DM commonly occurred post‐consultation in the community and typically related to side effects or medication costs. Such DM may be related to poor communication within the clinical consultation [89, 90]. This, therefore, raises the question of whether the DM continuum should capture the post‐consultation period. Most patients wanted or had some involvement in discussions about medication‐related DM. Over time, there has been an increasing shift towards older people taking a more proactive approach in discussions about their medication management. It was unclear if interventions have an impact on DM; however, they had a positive impact on collaborative discussion. Asking questions and having a companion present promoted involvement in SDM. However, there were often inconsistencies between patients and HCPs in how they perceived DM related to medications. A limitation in the evidence was that the majority of medication‐related decisions were related to prescribing (e.g., options, new medication, change in medication, ceasing a medication, dose change or formulation change). Few papers have reported on decisions related to other areas of medication management (such as accessing medications, when to take medications to meet patient preferences, cheaper brands and promoting adherence strategies).

#### 4.1.1 Lack of Definitional Clarity of SDM in Medication Consultations

All included papers comprised studies on communication between the HCP and patient and/or carer about medication management whereby the researchers observed, listened to or questioned participants about their involvement in at least one element of SDM. It was highlighted in Section 3.2 that seven papers provided a definition of SDM [15, 28, 51, 52, 53, 54, 55]. Each definition was different; however, it had similarities, with the focus of the definition being on a collaborative information exchange to reach a consensus on treatment. The rest of the authors did not provide a definition of SDM. It is, therefore, not surprising that the themes in this systematic review showed wide variation in DM related to medication management. With the ever‐increasing research being undertaken on SDM, and the lack of consistency in describing the concept [91], confusion will abound, especially for the reader [92] and fellow researchers [93]. Providing a definition of SDM, whether the authors' own or cited, to guide research will promote clarity in all steps of the research process necessary to develop sound SDM measures and readers will be able to clearly understand results [93].

#### 4.1.2 Variation in Operationalising SDM Measurement in Medication Consultations

This review revealed wide variation in the use of tools or questions to measure the SDM process and related outcomes, leading to heterogeneity in the findings [94]. Across the included studies, there were 36 interviews/focus groups, 14 questionnaires, 5 video/audio recordings and 4 observational reports. A range of different tools were used (see Tables 1 and 2). The reliability and validity of tools were reported in some papers; however, two tools were modified [39, 40, 41], with the reliability and validity of the modified tool not reported. Whenever a tool is adapted for use in a new context, which is different to the context in which it was originally tested, the tools' psychometric properties should be tested [95] to ensure the tool is suitable for use and to prevent any bias in the results.

In the studies using interview methods, the researchers designed questions relating to communication and/or involvement in the SDM process to examine a component of SDM (Appendix C). While pilot testing and revisions to interview schedules were undertaken in many studies, it is unclear if such testing was undertaken in all included studies. Steps to ensure questions are understandable to patient and carer participants and measure the concept being studied are crucial [96]. This is consistent with the findings of other systematic reviews where a variety of measurement tools were identified to measure SDM and also found to lack reliability and validity. This may be related to the absence of a standard measure for SDM outcomes [1, 94].

#### 4.1.3 Variation in Decision‐Making

Decision‐making in this review ranged from unitary DM (HCP‐driven or patient‐driven) to SDM. This has been described previously by Makoul and Clayman [97] who liken it to a continuum, with the HCP and patient at either end of the continuum as leaders of the discussion and decision, and the shared role depicted at the centre of the continuum.

In many studies, older people preferred, deferred or felt forced to defer to HCPs in making medication‐related decisions. People aged 65 years or older were born when a paternalistic approach was the norm [23], and as patients, the HCP was considered akin to God [23, 98]. Patients believed that if they questioned HCPs, they were interrogating the HCPs' expertise and challenging their authority, and as a result, negatively influencing the patient–HCP relationship [15, 53, 65, 67, 68]. Many participants felt they could not participate in decision‐making due to a lack of knowledge or feeling vulnerable due to illness. Declining health status or increasing multi‐morbidity are associated with poorer outcomes for the older person, such as impaired functional status, a reduced quality of life and an increased use of health services [99]. Patients referring to their inpatient experiences highlighted that with surgery or declining health, being involved in medication DM tended to be more HCP‐driven [49, 52, 80]. This is supported by the literature, where patients in hospital inpatient settings often felt disempowered, unable to ask questions, provide preferences or were unable to participate in SDM due to an increasing illness acuity [91, 100]. This presents an asymmetrical relationship between the patient and HCP. The patient in a dependent role is faced with uncertainty and lacks knowledge, and the HCP is in the expert role [7, 10]. For patients, where communication is perceived as disempowering, the interaction could impact the patient's sense of self‐efficacy in managing their health and well‐being, including medications.

Similar to other reports in the literature [101], a minority of patients appeared to prefer an active role in patient‐driven DM. In many studies, patient‐driven decisions resulted from perceptions of medication ineffectiveness, experiences of adverse effects, or medication cost, and often occurred outside of the medication consultation [69, 75].

This review revealed that some older people did not wish to participate in making the final decision but desired information or involvement in the DM process [9, 102]. Some participants did not recognise that medication prescribing was a decision in which they could be involved. Patients who were more accepting of medication changes, as seen in provider‐driven DM, were older people and had a chronic condition that required medication long‐term [18, 49, 53, 103]. Consistent with the findings of systematic reviews on decision aids to promote SDM, this review had varying results regarding interventional tools [1, 104]; however, such tools may promote discussion between the HCP and patient [15, 43]. A clear explanation of their purpose may be necessary for the older person [86]. Similar to other reports in the literature regarding the use of question‐asking prompts, as seen in the ‘Ask Me 3’ [105] and ‘5 Questions’ [106] programmes, this review highlights that providing pre‐prepared questions before the clinic visit was essential for collaborative discussion. This may highlight the need for a specific aid related to medications in general, provided either before or during the clinic appointment, to prompt patients to ask questions that are personalised to their needs. Participants highlighted examples of receiving information about medications and management plans, asking questions if unsure and providing information about themselves to the HCP as a part of the process, with or without being a part of medication‐related decisions.

### 4.2 Limitations and Strengths

The included studies varied in methodology and measurements; therefore, data could not be statistically pooled for analysis. Our search strategy excluded non‐English language publications and, therefore, may have resulted in a loss of information about medication‐related DM practices in other languages. The review was not limited to any specific methodology or date, which was a key strength.

### 4.3 Conclusion

This systematic review shows that older people need the knowledge to assist in the self‐management of medications. This includes information gleaned during conversations with HCPs when a decision is being made about a medication or when a change in medication occurs. Participants preferred to be involved in the process, which may or may not include the final decision. The responsibility for engagement in a collaborative discussion where a decision is required should include everyone involved.

When undertaking research in SDM, it is important to define the term so that the elements being examined are clear. The need for a standardised framework of SDM and its elements is evident. When designing research, each element of SDM should be clearly articulated and defined, and tools used to measure SDM should be tested for validity and reliability. Further, to advance understanding of SDM, further investigation is required into patient and carer understanding and interpretation of the concept. Further research into supporting the patient–HCP collaboration is required. This could include identifying barriers and facilitators to patients asking questions, developing the skills of HCP to promote collaborative discussion and developing tools to enhance patient involvement, followed by randomised controlled trials to evaluate the tools.

## Author Contributions

**Deana M. Copley:** conceptualisation, methodology, formal analysis, investigation, data curation, resources, writing – original draft, writing – review and editing, project administration. **Elizabeth Manias:** conceptualisation, methodology, formal analysis, investigation, validation, writing – review and editing, supervision. **Vanessa Watkins:** formal analysis, investigation, validation, writing – review and editing. **Alison M. Hutchinson:** conceptualisation, methodology, formal analysis, investigation, validation, writing – review and editing, supervision.

## Conflicts of Interest

The authors declare no conflicts of interest.

## Supporting information

Supporting information.

Supporting information.

Supporting information.

## Data Availability

The data that support the findings of this study are available from the corresponding author upon reasonable request.
