# Supplementary material for: Communication Processes Related to Decision‐Making in Medication Management Between Healthcare Providers, Older People and Their Carers: A Systematic Review
Source: Health Expect. 2025 Apr 20;28(2):e70252. doi: 10.1111/hex.70252 (PMC12010048; doi:10.1111/hex.70252)
Supplement: Supplementary file 1 — Supporting information. [file HEX-28-e70252-s001.docx]

**Appendix A**

Example search strategy using MEDLINE Complete

| # | Query | Limiters |
| --- | --- | --- |
| S5 | S1 AND S2 AND S3 AND S4 | Limiters - English Language; Human |
| S4 | TI (prescriber* OR nurs* OR practitioner* OR clinician* OR pharmacist* OR doctor* OR "general practitioner*" OR physician* OR "health professional*" OR "healthcare professional*" OR "health care professional*" OR "medical professional*" OR "health care provider*") OR AB (prescriber* OR nurs* OR practitioner* OR clinician* OR pharmacist* OR doctor* OR "general practitioner*" OR physician* OR "health professional*" OR "healthcare professional*" OR "health care professional*" OR "medical professional*" OR "health care provider*") |  |
| S3 | MH “decision making+” OR MM "patient participation" OR TI (((patient* OR consumer*) AND (attitude* OR participating OR participation OR involving OR involvement)) OR communication OR ((decision* OR choice*) AND (making OR support* OR behav*)) OR ((share* OR sharing OR informed) AND (decision* OR deciding OR choice*)) OR ((decision* OR choice*) N1 (making OR support* OR shared OR sharing OR inform* OR aid* OR behav*))) OR AB (((decision* OR choice*) N1 (making OR support* OR shared OR sharing OR inform* OR aid* OR behav*)) OR communication OR ((patient OR consumer) N1 (attitude* OR preference* OR participation OR involvement))) |  |
| S2 | MH("aged+" OR "aged, 80 years and over") |  |
| S1 | MH “pharmaceutical preparations+” OR TI ((decision* OR prescription* OR management OR treatment* OR therapy OR prescrib*) N4 (medicine* OR medication* OR drug*)) OR AB ((decision* OR prescription* OR management OR treatment* OR therapy OR prescrib*) N4 (medicine* OR medication* OR drug*)) |  |
