# Supplementary material for: Communication Processes Related to Decision‐Making in Medication Management Between Healthcare Providers, Older People and Their Carers: A Systematic Review
Source: Health Expect. 2025 Apr 20;28(2):e70252. doi: 10.1111/hex.70252 (PMC12010048; doi:10.1111/hex.70252)
Supplement: Supplementary file 2 — Supporting information. [file HEX-28-e70252-s003.docx]

| **Appendix B**: Quality Assessment using the Caldwell Framework | | | | | |  |  |  |  |  |  |  |  |  |  |  |  |  |  |
| --- | --- | --- | --- | --- | --- | --- | --- | --- | --- | --- | --- | --- | --- | --- | --- | --- | --- | --- | --- |
| **Author (s), (Year), Ref** | Does the title reflect the content? | Are the authors credible | Does the abstract summarise the key components? | Is the rationale for undertaking the research clearly outlined? | Is the literature review comprehensive & up-to-date? | Is the aim of the research clearly stated? | Are all ethical issues identified & addressed? | Is the methodology identified & justified? | **Quantitative** | Is the design clearly identified  and rationale provided? | Is there an experimental hypothesis clearly stated and are the key variable identified? | Is the population identified? | Is the sample adequately described and reflective of the population? | Is the method of data collection valid and reliable? | Is the method of data analysis credible and confirmable? | Are the results presented in a way that is appropriate & clear? | Is the discussion comprehensive? | Are the results generalisable? | Is the conclusion comprehensive? |
|  |  |  |  |  |  |  |  |  | **Qualitative** | Are the philosophical background  & study design identified & the rationale for choice of design evident? | Are the major concepts identified? | Is the context of the study outlined? | Is the selection of participants described & the sampling method identified? | Is the method of data collection auditable? | Is the method of data analysis credible & confirmable? |  |  | Are the results transferable |  |
| Andreas et al. (2010)^51^ | Yes | Yes | Yes | Yes | Yes | Yes | Yes | Yes | Qualitative | Yes | Yes | Yes | Yes | Yes | Yes | Yes | Yes | Yes | Yes |
| Belcher et al. (2006)^15^ | Yes | Yes | Yes | Yes | Yes | Yes | Yes | Yes | Qualitative | Yes | Yes | Yes | Yes | Yes | Yes | Yes | Yes | Yes | Yes |
| Bell et al. (2017)^70^ | Yes | Yes | Yes | Yes | Yes | Yes | Partly | Yes | Qualitative | Partly | Yes | Yes | Yes | Yes | Yes | Yes | Yes | Yes | Yes |
| Brünn et al. (2021)^58^ⱡ | Yes | Yes | Yes | Yes | Yes | Yes | Partly | Yes | Qualitative | Yes | Yes | Yes | Yes | Yes | Yes | Yes | Yes | Yes | Yes |
| Campbell et al. (2020)^88^ | Yes | Yes | Yes | Yes | Yes | Yes | Yes | Yes | Qualitative | Yes | Yes | Yes | Yes | Yes | Yes | Yes | Partly | Yes | Yes |
| Caughey et al. (2020)^71^ | Yes | Yes | Yes | Yes | Yes | Yes | Yes | Yes | Mixed | Yes | Yes | Yes | Partly | Yes | Partly | No | Yes | Yes | Yes |
| Dooley et al. (2019)^86^ | Yes | Yes | Yes | Yes | Yes | Yes | Yes | Yes | Qualitative | Yes | Yes | Yes | Yes | Yes | Yes | Yes | Yes | No | Yes |
| Eassey et al. (2017)^56^ⱡ | Yes | Yes | Yes | Yes | Yes | Yes | Yes | Yes | Qualitative | Yes | Yes | Yes | Yes | Yes | Yes | Yes | Yes | Yes | Yes |
| Fabricius et al. (2021)^52^ | Yes | Yes | Yes | Yes | Yes | Yes | Yes | Yes | Qualitative | Yes | Yes | Yes | Partly | Yes | Yes | Yes | Yes | Yes | Yes |
| Farrell et al. (2020)^64^ | Yes | Yes | Yes | Yes | Yes | Yes | Yes | Yes | Qualitative | Yes | Yes | Yes | Yes | Yes | Yes | Yes | Yes | Yes | Yes |
| Fried et al. (2017)^39^# | Yes | Yes | Yes | Yes | Yes | Yes | Partly | Yes | Quantitative | Partly | Partly | Yes | Partly | Yes | Yes | Yes | Yes | No | Yes |
| Gillespie et al. (2019)^40^* | Yes | Yes | Yes | Yes | Yes | Yes | Yes | Yes | Quantitative | Yes | Yes | Yes | Yes | Partly | Yes | Yes | Yes | No | Yes |
| Gillespie et al. (2022)^42^* | Yes | Yes | Yes | Yes | Yes | Yes | Yes | Yes | Qualitative | Yes | Yes | Yes | Yes | Yes | Yes | Yes | Yes | Yes | Yes |
| Gillespie et al. (2023)^41^* | Yes | Yes | Yes | Yes | Yes | Yes | Yes | Yes | Mixed | Yes | Yes | Yes | Yes | Yes | Yes | Yes | Yes | Yes | Yes |
| Green et al. (2020)^87^ | Yes | Yes | Yes | Yes | Yes | Yes | Yes | Yes | Qualitative | Yes | Yes | Yes | Partly | Yes | Yes | Yes | Yes | Yes | Yes |
| Haverhals et al. (2011)^65^ | Yes | Yes | Yes | Yes | Yes | Yes | Partly | Yes | Qualitative | No | Yes | Yes | Yes | Yes | Yes | Yes | Yes | Yes | Yes |
| Højgaard et al. (2024)^78^ | Yes | Yes | Yes | Yes | Yes | Yes | Yes | Yes | Qualitative | Yes | Yes | Yes | Yes | Yes | Yes | Yes | Yes | Yes | Yes |
| Holmqvist et al. (2019)^59^ | Yes | Yes | Yes | Yes | Yes | Yes | Yes | Yes | Qualitative | Yes | Yes | Yes | Yes | Yes | Yes | Yes | Yes | Yes | Yes |
| Hu et al. (2023)^55^ | Yes | Yes | Yes | Yes | Yes | No | Yes | Yes | Quantitative | Yes | Yes | Yes | Yes | Yes | Yes | Yes | Yes | No | Yes |
| Jansen et al. (2019)^53^ | Yes | Yes | Yes | Yes | Yes | Yes | Yes | Yes | Qualitative | Yes | Yes | Yes | Yes | Yes | Yes | Yes | Yes | Yes | Yes |
| Junius-Walker et al. (2021)^63^ | Yes | Yes | Yes | Yes | Yes | Yes | Yes | Yes | Quantitative | Yes | Yes | Yes | Partly | Yes | Yes | Yes | Yes | Yes | Yes |
| Kempen et al. (2020)^80^ | Yes | Yes | Yes | Yes | Yes | Yes | Yes | Yes | Qualitative | Yes | Yes | Yes | Yes | Yes | Yes | Yes | Yes | No | No |
| Knight et al. (2013)^79^ | Yes | Yes | Yes | Yes | Yes | Yes | Yes | Yes | Qualitative | Yes | Yes | Yes | Yes | Yes | Yes | Yes | Yes | Yes | Yes |
| Kreling et al. (2006)^66^ | Yes | Yes | Yes | Yes | Yes | Yes | Yes | Yes | Qualitative | No | Yes | Yes | Yes | Yes | No | Yes | Yes | Yes | Yes |
| Lansbury (2000)^62^ | Yes | Yes | Yes | Yes | Yes | Yes | Yes | Yes | Qualitative | Yes | Yes | Yes | Yes | Yes | Yes | Yes | Yes | Yes | Yes |
| Manias et al. (2024)^50^ | Yes | Yes | Yes | Yes | Yes | Yes | Yes | Yes | Qualitative | Yes | Yes | Yes | Yes | Yes | Yes | Yes | Yes | Yes | Yes |
| McCabe et al. (2019)^28^ | Yes | Yes | Yes | Yes | Yes | Yes | Yes | Yes | Qualitative | Partly | Yes | Yes | Yes | Yes | Yes | Yes | Yes | Yes | Yes |
| **Author (s), (Year), Ref** | Does the title reflect the content? | Are the authors credible | Does the abstract summarise the key components? | Is the rationale for undertaking the research clearly outlined? | Is the literature review comprehensive & up-to-date? | Is the aim of the research clearly stated? | Are all ethical issues identified & addressed? | Is the methodology identified & justified? | **Quantitative** | Is the design clearly identified  and rationale provided? | Is there an experimental hypothesis clearly stated and are the key variable identified? | Is the population identified? | Is the sample adequately described and reflective of the population? | Is the method of data collection valid and reliable? | Is the method of data analysis credible and confirmable? | Are the results presented in a way that is appropriate & clear? | Is the discussion comprehensive? | Are the results generalisable? | Is the conclusion comprehensive? |
|  |  |  |  |  |  |  |  |  | **Qualitative** | Are the philosophical background& study design identified & the rationale for choice of design evident? | Are the major concepts identified? | Is the context of the study outlined? | Is the selection of participants described & the sampling method identified? | Is the method of data collection auditable? | Is the method of data analysis credible & confirmable? |  |  | Are the results transferable |  |
| Mc Gillicuddy et al. (2019)^67^ | Yes | Yes | Yes | Yes | Yes | Yes | Yes | Yes | Qualitative | Yes | Yes | Yes | Yes | Yes | Yes | Yes | Yes | Yes | Yes |
| Mecca et al. (2022)^43^# | Yes | Yes | Yes | Yes | Yes | Yes | Yes | Yes | Mixed | Yes | Yes | Yes | Partly | Yes | Yes | Yes | Yes | Yes | Yes |
| O'Quinn et al. (2015)^82^ | Yes | Yes | Yes | Yes | Yes | Yes | Partly | Yes | Qualitative | Yes | Yes | Yes | Yes | Yes | Yes | Yes | Yes | Yes | Yes |
| Ouellet et al. (2022)^72^ | Yes | Yes | Yes | Yes | Yes | Yes | Yes | Yes | Qualitative | Yes | Yes | Yes | Yes | Yes | Yes | Yes | Yes | No | No |
| Parekh et al. (2019)^81^ | Yes | Yes | Yes | No | No | Yes | Partly | Yes | Qualitative | Yes | Yes | Yes | Yes | Yes | Yes | Yes | Yes | Yes | Yes |
| Peat et al. (2023)^57^ | Yes | Yes | Yes | Yes | No | Yes | Partly | Yes | Qualitative | Partly | Yes | Yes | Yes | Yes | Yes | Yes | Partly | Partly | No |
| Perreira, Bieri, del Rio Carral et al. (2022)^45^$ | Yes | Yes | Yes | No | Yes | Yes | Yes | Yes | Qualitative | Yes | Yes | Yes | Yes | Yes | Yes | Yes | Yes | No | Yes |
| Perreira, Bieri, Martins et al. (2022)^44^$ | Yes | Yes | Yes | No | Yes | Yes | Yes | Yes | Qualitative | Yes | Yes | Yes | Yes | Yes | Yes | Yes | Yes | No | Yes |
| Reeve et al. (2016)^73^ | Yes | Yes | Yes | Yes | Yes | Yes | Yes | Yes | Qualitative | No | Yes | Yes | Yes | Yes | Yes | Yes | Yes | Yes | Yes |
| Ross & Gillett (2021)^74^ | Yes | Yes | Yes | Yes | Yes | Yes | Yes | Yes | Qualitative | Yes | Yes | No | No | No | No | No | Yes | Yes | Yes |
| Sale et al. (2011)^75^ | Yes | Yes | Yes | Yes | Yes | Yes | Yes | Yes | Qualitative | Yes | Yes | Yes | Yes | Yes | Yes | Yes | Yes | Yes | Yes |
| Salter et al. (2014)^83^ | Yes | Yes | Yes | Yes | Yes | Yes | Yes | Yes | Qualitative | Yes | Yes | Yes | Yes | Yes | Yes | Yes | Yes | Yes | Yes |
| Schmittdiel et al. (2010)^85^ | Yes | Yes | Yes | Yes | Yes | Yes | Yes | Yes | Quantitative | No | Yes | Yes | Yes | Yes | Yes | Yes | Yes | No | Yes |
| Schopf et al. (2018)^68^ | Yes | Yes | Yes | Yes | Yes | Yes | Yes | Yes | Qualitative | Yes | Yes | Yes | Yes | Yes | Yes | Yes | Yes | Yes | Yes |
| Smith et al. (1994)^60^ | Yes | Yes | Yes | Yes | Yes | Yes | No | Yes | Quantitative | Yes | Yes | Yes | No | Yes | Yes | Yes | Yes | No | Yes |
| Spinewine et al. (2005)^76^ | Yes | Yes | Yes | Yes | Yes | Yes | Yes | Yes | Qualitative | Yes | Yes | Yes | Yes | Yes | Yes | Yes | Yes | Yes | Yes |
| Thevelin et al. (2022)^49^ | Yes | Yes | Yes | Yes | Yes | Yes | Yes | Yes | Mixed | Yes | No | Yes | Yes | Yes | No | Yes | Yes | Yes | No |
| Tietbohl & Bergen (2022)^77^ | Yes | Yes | Yes | Yes | Yes | Yes | Yes | Yes | Qualitative | Yes | Yes | No | Yes | No | Yes | Yes | Yes | Yes | Yes |
| Tinetti et al. (2024)^61^ | Yes | Yes | Yes | Yes | Yes | Yes | Yes | Yes | Quantitative | Yes | Yes | Yes | Yes | Yes | Yes | Yes | Yes | Yes | Yes |
| Tinetti et al. (2019)^47^ | Yes | Yes | Yes | Yes | Yes | Yes | Yes | Yes | Quantitative | Yes | Yes | Yes | Yes | Yes | Yes | Yes | Yes | Yes | Yes |
| Tjia et al. (2008)^69^ | Yes | Yes | Yes | Yes | Yes | Yes | Yes | Yes | Qualitative | Yes | Yes | Yes | Yes | Yes | Yes | Yes | Yes | Yes | Yes |
| Tobiano et al. (2021)^27^ | Yes | Yes | Yes | Yes | Yes | Yes | Yes | Yes | Mixed | Yes | Yes | Yes | Yes | Yes | Yes | Yes | Yes | Yes | Yes |
| Weir et al. (2021)^54^ | Yes | Yes | Yes | Yes | Yes | Yes | Yes | Yes | Qualitative | Yes | Yes | Yes | Yes | Yes | Yes | Yes | Yes | Yes | Yes |
| Weir et al. (2018)^46^ | Yes | Yes | Yes | Yes | Yes | Yes | Yes | Yes | Qualitative | Yes | Yes | Yes | Yes | Yes | Yes | Yes | Yes | Yes | Yes |
| Wilson et al. (2007)^84^ | Yes | Yes | Yes | Yes | Yes | Yes | Yes | Yes | Quantitative | Yes | Yes | Yes | Yes | Yes | Yes | Yes | Yes | Yes | Yes |
| Xu et al. (2003)^48^ | Yes | Yes | Yes | Yes | Yes | Yes | No | Yes | Quantitative | Yes | Yes | Yes | Yes | Yes | Yes | Yes | Yes | Yes | Yes |
| * Articles reporting the same study |  |  |  |  |  |  |  |  |  |  |  |  |  |  |  |  |  |  |  |
| # Articles reporting the same study |  |  |  |  |  |  |  |  |  |  |  |  |  |  |  |  |  |  |  |
| Articles reporting the same study |  |  |  |  |  |  |  |  |  |  |  |  |  |  |  |  |  |  |  |
