# Supplementary material for: Communication Processes Related to Decision‐Making in Medication Management Between Healthcare Providers, Older People and Their Carers: A Systematic Review
Source: Health Expect. 2025 Apr 20;28(2):e70252. doi: 10.1111/hex.70252 (PMC12010048; doi:10.1111/hex.70252)
Supplement: Supplementary file 3 — Supporting information. [file HEX-28-e70252-s002.docx]

| **Appendix C: Study Questions and Results** | |  |
| --- | --- | --- |
| **Author (s), (Year), Country, Ref** | **Questions asked/Topic guide in study related to SDM** | **Descriptive Results / Supporting Participant Quotes** |
| Andreas et al. (2010) US^51^ | Topic guide:  Participants’ beliefs regarding (a) burdens, (b) responsibilities, (c) adverse events, and (d) benefits of their medication regimens Detailed prompts to probe patients’ expectations of participation in decision making and beliefs regarding the balance of managing bothersome symptoms and treating asymptomatic chronic conditions | "Those are some of the lessons why I learned, you ask . . . “Just will this match what I’m taking?”" (p. 1160) “As far as the doctor, uh, they don’t discuss too much about it, just say, “Well, I’m going to put you on this right here.” . . . And that’s it” (p.1160) "But you have to ask. . . . It’s your responsibility. “Does it match?” And the VA has got a sign up in there to ask the doctor if the medication that’s being prescribed to you matches your other things." (p.1160) "the question what percentage is it our responsibility? ... and I’m not being facetious … A hundred percent … you must report if you have a side effect. ... Don’t just say, “Oh, I’ll be fine,” because I learned a long time ago, if you don’t tell them, “Oh, it hurts like hell,” they don’t do anything." (p.1161) |
| Belcher et al. (2006) US^15^ | Questions: "Each person is different in how they talk to, and interact with, their doctor about medicine. How do you talk to the doctor about medications?" "What do you talk about?" "When making decisions about what medicine to give a patient, it helps doctors to know the patient's feelings and concerns about the medicine. What things can you think of that might help people be a part of making decisions about their medicine?" "What things keep people from being a part of making decisions about medicine with their doctor?" | ""I don't know anything about them. How can I make a decision?" "I have some friends that feel the doctor is still Almighty God, like they did umpteen years ago. The doctor prescribed and the patient took It whether she wanted to or not." (p.300) “ ... and you know, you should feel free to ask them whatever you want concerning yourself. You know your body, yourself, or your medication"; "the people should not be afraid to ask questions even if it means, like I do, I make a little list when I go in ... " (p.300) "It's sort of a trial and error thing, before you can find out which is the right one ... It was the way it hit me ... we had to go back and try something different." “It's taken two or three different medications to get it (hypertension) under control. But now it is under pretty good control” (p.300) " . . . he [the doctor] went along with it of course ... But I don't think he had thought enough about the, what's the word I'm looking for, ... well-being ... " (p.300) "Well I put my hands in my doctor's hands, and if he tells me this is good for me then I believe him." (p.300) "Well it all depends on how the doctor reacts. Some doctors are just so selfish you don't want to ask them nothing ... like they don't care, you know ... " (p.301) "So I don't know whether it is possible for a lay person to be involved in their medication unless the doctor sits down and says to them, well we have a choice of doing A, B, and C, And then outlines what A, B, and C are, and then offers them a choice." (p.301) |
| Bell et al. (2017) Norway^70^ | Questions: "Have you experienced dizziness or falls? Can you explain to me what happened?" "Can you tell me which medicines you are taking and what information you have received from your physician?" "Do you associate the use of your medicines with dizziness and falls? Please explain" | "I have just accepted it, because I fully trust my GP. I do not have an opinion (…) because that is up to the GP to decide. It is his responsibility." (p. 251).  "When the hospital physician called me back I told him that the metoprolol is affecting my quality of life and make me anxious about when the dizziness will emerge. (…) He understood me and said ‘that is why we need to do something about it’. He told me I could reduce the dose to 25 mg, but it was up to me. (…) I want to discuss it with my GP first so that I don’t have to make the decision entirely on my own." (p. 252) |
| Brünn et al. (2021) Germany^58^ⱡ | Questions: "What happens when you suggest discontinuing a medication to your GP?" "What happens when your GP suggests you discontinue a medication?" "Are you taking a medication you don’t really want to take?" "Tell us what happened when your GP last spoke to you about your medication?" | “I noticed that I feel much better when I don’t take [a specific drug]. I told [the GP] and since then, we have stopped it.” ([P2]p.5) “We tried twice. I asked [the GP] if father really needed the diuretic […] and each time […] after checking the lab results, she said: ‘No, he needs it for such and such a reason.’” ([Daughter of P4/P5 ] p.5-6) “I realized [my blood pressure] was very low. […] [My GP and I] talked about it and realized I was taking half a tablet too much. […] But we measured my blood pressure for several days first. […] And once, […] after taking a blood sample, the GP said […] my cholesterol level was normal again. […] Then we reduced [the medication].” ([P7]p.6) “I have considerable trust in my GP. When he says: ‘You should take this’, […] I assume it to be true. […] I think it’s always better when people discussing things are experts rather than laypersons. And when my GP explains things afterwards, I understand it, too.” ([P7]p.6) “I asked [my GP]: ‘Can we deprescribe something?’ Because it is a lot. And he replied: ‘Actually, you need all the drugs.’ And that was that as far as I was concerned.” ([P6]p.6) |
| Campbell et al. (2020) Canada^88^ | Patient questions: "Did you take the letters you received from Moxie to your family doctor or pharmacist?" "If no, why did you not bring them to your healthcare provider?" Depending on whether patient commenced on medication, prompted with:  (1) "If yes, did you have a discussion around starting this new medication?" "If yes, why did you ultimately decide to not start the medication?" (2) "If yes, were these helpful?" "If no, why not?" "If yes, how so?" Pharmacist questions: "Did any conversations about statins/ACE-ARBs start because of this letter?" "Did you ever fax the recommendations to the patient's prescriber?" "Did any patients get started on one of these medications because of this letter?" | Descriptive: Participants receiving a letter from Moxie to commence a statin (n=24); Participants taking letter to HCP (n=15): No Discussion (n=8) Quotes:  "I gave the one to the doctor, but he said, no that's alright, you keep it." "I don't even know because I just gave him the envelope and didn't really get a response from them." (p.1490) '"I just give him the letter and he just looked at it and put it in the file I guess." "He looked at them and he said – no you are ok, you're ok, we will just leave these alone." (p. 1491) "I had to tell him, I think we should do it. So, then he gave me a smaller dose". (p. 1491) |
| Caughey et al. (2020) Australia^71^ | Patient topics: "i) treatment preferences, ii) discussion of medication adverse effects and iii) SDM" HCP Topics  "i) their overall approach to treatment DM for older patients with multimorbidity, ii) patient preferences and iii) polypharmacy" | Descriptive - Participant: 60% (n=9) said they regularly discussed their treatment preferences with their HCP. Of these, 22% (n=2) stated they would defer to the HCP decision, and 22% (n=2) stated they thought their HCP did not encourage such discussions. 66.7% (n=10) of participants stated they recalled their HCP discussing risks and benefits of medications. 33.3% (n=5) recalled receiving information about risks or benefits or nothing. All participants indicated they would like more information about medications.  66.7% (n=10) of participants did not want greater involvement in SDM. The remaining 33.3% (n=5) who wished to have more involvement in SDM, however felt it was unrealistic. Descriptive - HCP: Treatment DM based on 2 factors: (1) prognosis, and (2) improving quality of life by relieving symptoms. |
| Dooley et al. (2019) UK^86^ | Treatment Recommendation Coding Scheme:  Coded on 5 formats - pronouncements, proposals, suggestions, offers, and assertions | Descriptive and Quotes: 71 recommendations for medication were made. 73% (n=52) of treatment discussions were initiated with assertions, with 13% (n=9) as the only treatment recommendation (e.g. information giving), "Now there is some medication that many people take to help with the symptoms of memory loss" (p.215). Assertions were met with acceptance (n=2) or passive resistance (n=7) by patients with dementia.  42% (n=30) of consultations used suggestions, "Would you like to think about taking some medication?" (p.215). Suggestions were met with acceptance (n=2) or passive / active resistance (n=27) by patients with dementia.  25% (n=18) of consultations used proposals, "We could try you on a tablet to help contain or maintain or make this stable in the future" (p.215). Proposals were met with acceptance (n=7) or passive / active resistance (n=11) by patients with dementia.  11% (n=8) of consultations used pronouncements, "I would want you to start at least taking medication" (p.215). All pronouncements were met with passive resistance by patients with dementia.  9% (n=6) of consultations used offers, "The other thing we can do is to give you a tablet if you would like?" (p.215). Offers were met with acceptance (n=1) or passive resistance (n=5) by patients with dementia. |
| **Author (s), (Year), Country, Ref** | **Questions asked/Topic guide in study related to SDM** | **Descriptive Results / Supporting Participant Quotes** |
| Eassey et al. (2017) Australia^56^ⱡ | Question:  "What are your opinions about the role of the consumer/patient in managing medicines? For example, should consumers play a role in deciding which medicines and strengths are prescribed? Should the doctor make all the decisions?" | "[I] started insulin without proper directions given also [there was a] change to pills… [I] only found out after getting home" (66-year-old) (p.1117) "[I’m] not very happy since it (swollen breasts) was a side effect of taking Duodart (dutasteride and tamsulosin) and the doctor didn’t tell me about the side effect" (69-year-old) (p.1117) "I trust my doctor so he/she should make those [medications] decisions for me" (68-year-old) (p.1117) "…I am not a doctor and believe that they know/understand my conditions better than me" (77-year-old) (p.1117) "The consumer should have the right to have some input into the medications they are prescribed. In my case my doctor prescribed a certain strength blood pressure medication that caused bad headaches for me… we worked out a compromise of taking a lower dose one day and the stronger one on the alternative day…" (67-year-old) (p.1117) "I was consulted by the doctor about my medicines & my opinions were taken into consideration, but I can see that patients with mild Dementia or other mental issues would need firm leading" (78-year-old) (p.1117) |
| Fabricius et al. (2021) Denmark^52^ | Questions: "Do you involve patients in their medication?" "Why/why not?" "At what point in the process?" "Why?" "Are there any situations when the physician/pharmacist/nurse decide without involving the patient?" "Which are the biggest barriers in patient involvement in the medication process?" "Which are the biggest opportunities?" "What is meant by the term “unmanageable” medication?" Do you see any connections between "unmanageable medication" (or the number of drugs) and the possibility of patient involvement?" | Descriptive and Quotes: Themes evolved which influenced patient involvement: these related to the (1) blurred roles of different HCPs:  ED doctors did not discuss medications with patients as they believed this was the patient's GP's role. Nurses would discuss medications with patients and then pass this information onto the prescriber. (2) Older person and the complexity of polypharmacy: Some prescribers were reluctant to trust older people's accounts of their medications and therefore did not involve them in decisions; "What can you expect from an 87-year-old?" (p.10).  (3) time: One HCP indicated that “The physicians need to encourage the patient because the patient will not speak up unless we ask them" (p.11), however, asking questions of older people was seen as a challenge and takes time. |
| Farrell et al. (2020) Canada^64^ | Questions: "How do you like to learn about your medications-who usually does the teaching?" "Have you ever had questions about your medications? How did you get them answered?" "How do you feel about the medication change you had while at the Day hospital?" Probes: "What was that process like?" "Did you learn anything new about your medications while you were there?" "How have the changes gone? (e.g. process, impact?)" New prescription: "What did the person who prescribed it say at that time about their impression of the situation?" Probes: "What was the patient told?" "Whether a discussion about potential causes (i.e., drug side effect) occurred" "What options were discussed for management?" | “I was really upset when I was in the hospital, I didn’t remember really what the doctor told me about everything” (p.6) “I just take whatever I take and I don’t worry about it now. I know that sounds pretty terrible. It wasn’t always this way” (p.6) “My thinking was, if this is causing this accumulation of water in the ankles, it is not desirable so I made an appointment and he agreed” (p.7) “I keep thinking it must be the medication, but people won’t listen to me… Maybe because I don’t ask the questions, I don’t get the answers” (p.7) “The patient has to communicate and be forthcoming, otherwise, how can they make decisions, for example family doctors, I am not the only one, she has hundreds of them, so unless I tell her, how will she know?” (p.7) |
| Fried et al. (2017) US^39^# | Patient Assessment of Chronic Illness Care (PACIC):  Subscales relevant to SDM: patient activation, goal setting, and problem-solving and contextual counselling (Scores ranged from 0 to 12) Active patient Participation Coding Scheme:  Identifies (1)Three types of speech (questions, assertive responses, and expressions of concern) and (2) Two types of clinician communication (partnership-building responses and supportive talk) Counting utterances related to medication side effects, instructions, assistance with administration, purpose, regimen complexity , and new or changes in medications | Descriptive: PACIC: 29.7% of patients receiving Tool to Reduce Inappropriate Medications versus 15.6% of the control group scored 11/12 (P = 0.6, not significant) Bivariate analysis: patients receiving the intervention had more active participation (mean: 5.6 vs 2.7. P=0.001) and medication-related communication (mean: 7.5 vs 3.6. P=<0.001) than their control counterparts. HCPs of patients receiving the intervention were more facilitative (mean: 1.53 vs 0.67; P=0.02) and had more medication-related communication (mean: 7.3 vs 4.6. P=0.002) than HCPs of patients in the control group. Multivariate analysis - The results of this study found that the more engaged the patient was when communicating with their HCP, the more facilitative the HCP was. |
| Gillespie et al. (2019) Australia^40^* | Adapted Canadian Survey of Experiences with Primary Health Care: 1. "In the past 12 months, how often did your doctor involve you in decisions related to your medicines?" 2. "Does your doctor allow you enough time to discuss your feelings, fears or concerns about new medicines or medicines you may have been taking for some time?" | Descriptive: Adapted Canadian Survey of Experiences with Primary Health Care: 1. Rarely - 15.3% (n=21); Sometimes - 30% (n=41); Often - 41.6% (n=57) 2. Rarely - 9.5% (n=13); Sometimes - 28.5% (n=39); Often - 53.3% (n=73) |
| Gillespie et al. (2022) Australia^42^* | Older person questions: "Thinking about a typical visit to your regular GP, what things do you discuss with your doctor about your medications?" Probes: "What questions do you like to ask about your medications?" "What information do you like to know about your medicines?" "If there needs to be changes to your medication, how does your doctor involve you in making a decision about this?"  GP questions: "Could you tell me the principles that guide your prescribing practice for your community living older patients?" | Themes & Quotes: Views of ageing: "When you give an instruction, they may not really understand it" (GP, p.e6209) "So it's also what the person wants as well as what I want and so we negotiate something that we can both live with… Explain the good effects and the bad effects of a medicine to the person, their likelihood of getting good effects, their likelihood of getting bad effects, and then seeing what they want" (GP, p.e6210)  "And you have the conversation and as we're getting older, we forget" (Older adult, p.e6210)  "I've changed GPs in the beginning of the year because I wasn't happy with the GP I was seeing. Everything as far as she was concerned was down to age…. I didn't feel I was ready to be discarded quite yet" (Older adult, p.e6210) Trust: "I take my—whatever they've given me -it's trust really, a trust issue. It's a definitely trust issue, because if I wouldn't trust these doctors, I probably would have questioned it more often" (Older adult, p.e6210) "(they) were bought up in…a culture of respect for GPs" (GP, p.e6211) Limited Knowledge: "I trust that he knows best because I'm not a medical man, and I –never tried to be" (Older adult, p.e6211) ""Well, I don't want you to take Lisinopril anymore”…I didn't like that idea, but I did it and it didn't work. So, I didn't say too much… I just said, “All right then." So, yeah, they're the doctor. I'm not a doctor… I'm not one to interfere with things that I shouldn't interfere with" (Older adult, p.e6211) "Well…you pick up Google, don't you? And you look for reliable –as far as you know –thing and read it. But it gives me the basis for asking a question…. Not believing everything they say by any means, but giving me a bit more understanding" (Older adult, p.e6211) Participating in Medication Reviews: "We talk about the medications . . . she sort of runs through them all, but she doesn’t necessarily talk about them all, you know what I mean? I think she just sort of seems to accept that if things are going okay that we’ll stick with that" (Older adult, p.e6211) Health System: "I think they write it and they tell you, you need to be taking this. I mean –and I think they're time-poor. They're pushing you through, so you really don't get very much information" (Older adult, p.e6212) |
| Gillespie et al. (2023) Australia^41^* | GP survey questions -  1. "Overall, I believe my older adult patients can engage in a decision making process regarding medication continuation or deprescribing" 2. "I always ask my older adult patients what their preference is regarding medication continuation or deprescribing" 3. "Most of my older adult patients prefer to make decisions on their own about their medications after seriously considering my opinion" 4. "My older adult patients prefer to share responsibility for making medication decisions with me" 5. "My older adult patients prefer me to make their medication decisions for them" Adapted Canadian Survey of Experiences with Primary Health Care: "In the past 12 months, how often did your doctor involve you in decisions related to your medicines?" "Does your doctor allow you enough time to discuss your feelings, fears or concerns about new medicines or medicines you may have been taking for some time?" | Descriptive: Phase 1- GP survey questions -  1. Agree 84.7% (n=72); Neutral 12.9% (n=11); Disagree 2.4% (n=2) 2. Agree 64.7% (n=55); Neutral 22.4% (n=19); Disagree 10.6% (n=9) 3. Agree 12.9% (n=11); Neutral 40% (n=34); Disagree 45.9% (n=39) 4. Agree 63.5% (n=54); Neutral 17.6% (n=15); Disagree 17.6% (n=15) 5. Agree 49.4% (n=42); Neutral 36.5% (n=31); Disagree 12.9% (n=11) |
| **Author (s), (Year), Country, Ref** | **Questions asked/Topic guide in study related to SDM** | **Descriptive Results / Supporting Participant Quotes** |
| Green et al. (2020) US^87^ | Deductive Coding using SDM Framework:  Patient role in DM, discussion of problem, options, benefits and risks or any uncertainties with the medication, assessment of patient understanding, exploring patient preferences | "Now we have an event that is happening [i.e., falls], we need to modify. Down the road, if I want to take you off of the amitriptyline and replace it with something for neuropathy, would you be okay with that?" (p.239) "I want to get to know you a little bit more…And then work together on minimizing your medications. Understanding that I need to take care of your neuropathy, I need to make sure you're sleeping okay, and I need to make sure your blood pressure is okay. So I will not sacrifice what needs to be done, but I will try to minimize the amount of medicine that we use." (p. 240) |
| Haverhals et al. (2011) US^65^ | Questions:  "One challenge we’re especially interested in is keeping track of multiple medicines and making sure you’re taking the right ones at the right times. I’d be interested to hear how you do that." Probes inc: asking questions, taking notes, monitoring, keeping track of regimens etc.  "If you have questions about your medicines, what do you do to get those questions answered?" "Does your doctor, pharmacist or both explain your new meds if you are on them?" "What information about your medicines is most useful for you?" "Does your doctor give you any written instructions for taking your medicines when he or she prescribes them for you? If not, how do you remember what your doctor tells you after you have left an office visit?" "Do you keep track of your medicines? How do you keep track of what to take and when?" | "For instance, this doctor had prescribed medication for me and I started taking it and I was disturbed because I woke up in the middle of the night and my mouth was so dry and my throat was so dry and now I was having trouble with urination and I thought you know, that medication is doing this to me. And I’m stopping taking it." (p.5) "I have a lot of hesitancy with respect to medications and I feel like she is getting too much medication and more than likely some of her problems are caused by medication. So I’ve been attempting to modify her medications somewhat. Like weaning her off of prescribed depression medication and using the homeopathic medicine. So in a sense I am being somewhat of a diagnostician and dispenser." (p.7) "It’s just here take this, take this, take this ..." (p.8) |
| Højgaard et al. (2024) Denmark^78^ | Question & Topics: Significance & Meaning of recommending medication: "• When you think back to the conversation with the screening nurse, what were your thoughts when she recommended preventive medication? • What factors influenced your decision to take/not take the medication? • What was required for you to decide to take/not take the medication? • Did you have any concerns about taking the medication?" Support in Making an Informed Choice: "• How did you receive help in deciding whether to take the medication? • Who supported you? • Why was it helpful for you? • Was there anything you lacked in making the decision? • How do you feel about the information given regarding the medication? • Was the information relevant to you? • What is important for you to know about the medication? • Did you feel any information was missing?" | "I had a bad conscience about saying no to the medicine, but at the same time, I gradually believed in myself, so much that when I said, this is not good for me, I have to stop." (p.808 "We talked about the medication I might have to take, but since there was so little atherosclerosis and you could barely see it, I chose not to take it." (p.808) "I guess you believe in the authorities if they are health professionals who tell me that it will benefit me, then I'll take it." (p 809) "So, in the end, it's me who decides. It is my health, so I must take care of it. After all, no one else does. Because, otherwise, you could say that someone is pulling it down over your head." (p.809) "I think it's important to have your GP involved; it means something to me. With things like this, it's nice that it's someone who knows me. I trust that people who recommend it do so in good faith." (p.809) "So, I have to be able to sense that the information is for me and that there is a name on it." (p.809) |
| Holmqvist et al. (2019) Sweden^59^ | Question & Topics: "Describe your latest appointment with a physician, in which you talked about your medication treatment" - questions followed asking about how the medication treatment was evaluated, whose responsibility, participant safety, and participant involvement. | "Yes, as I was about to say… The responsibility must be mine almost. That I alert them if it would fail. However, otherwise, the responsibility is there [with the physician] to do it. Because they have not told me anything else" (p. 1297) "Yes, some things one remembers ‐‐‐ but it can be like stuffing too much information in, so to say. When you sit and go through a list like this, you know, and you concentrate ‐‐‐there may be something that gets lost, you know" (p.298) "Now, now that I got this plan from the doctor’s office on how they intended to do it. ‐‐‐ It is the first time they have reported what they have been thinking ‐‐‐ And how they have planned to manage it. Otherwise, it is just that, you get a new appointment and you get better. Bye! Send them off to home!"(p. 1299) "Because I know that when I got blood pressure medications then, ‐‐‐ then the doctor said ‘If there is anything that you feel then, that you have not felt before, because you are taking this medication, you will have to let us know’ ‐‐‐ But I have never felt that" (p.1300) "No, no, nothing, but she said ‘You should take this pill’ and I had no idea what it was. ‐‐‐ She could have sat down [bedside] and said that this one is for this and that and so on, but no… ‐‐‐ It’s not… they do not have time for that" (p.1300) |
| Hu et al. (2023) China^55^ | Questions: Control Preference Scale-Post: (participants chose the statement that best described what occurred in a communication with their HCP either when a new medication was prescribed or when a medication adjustment was made)  (1) I made my medication decision alone; (2) I made my medication decision alone considering what my doctor said; (3) I shared the medication decision with my doctor; (4) My doctor decided considering my preferences; (5) My doctor made the medication decision | Control Preference Scale-Post:   65-75 years (n=641) >75years (n=260) Patient involved DM (1 & 2) - 5.2% (n=33) 5.8% (n=15) SDM (3) - 36.8% (n=236) 31.9% (n=83) HCP-driven DM (4 & 5)- 58% (n=372) 62.3% (n=162) |
| Jansen et al. (2019) Australia^53^ | Questions: "Are you on any treatment for your heart/BP/cholesterol? If yes: a. What does the treatment involve?" Probes: "What did your doctor say to you when he/she recommended starting treatment?" "Why do you think your doctor recommended start treatment?" "How did you feel about starting treatment?"  "How much do you consider other illnesses or health problems that you may have in your decision-making about methods to reduce your heart disease risk?" "How do you prefer your doctor to make decisions about your heart disease risk and treatment - to what extent would you like to be involved?" | "Oh yes, I’d like to be completely involved, yes. INTERVIEWER: When you say completely involved you mean (…) PARTICIPANT: Getting as much information as what I can get" (p.4) "There are people who know, seem to know more about the pharmaceutical industry than the pharmacist. I’m not in that category, I do as I’m told" (p.4) "My muscles ached and I didn’t do well on them (a lipid lowering medication) and I hated taking them so… we’ve come to make a pact, as my HDLs and LDLs are a reasonable balance, I just go along with my high cholesterol which is monitored on a regular basis and lived with" (p.4) "[The GP] made sure that I understood what was going to happen (…), and she left the choice [to take medication for elevated blood pressure] to me" (p.5) “That’s what they’re there for, that’s their job. My job is to, to do what they tell me” (p. 5) |
| Junius-Walker et al. (2021) Germany^63^ | Topics: GP survey: involvement in the decision Patient questionnaire (Time 1): satisfaction with receiving and sharing information, involvement in the decision Patient questionnaire (Time 2): sustained satisfaction with the decision | Of the 37 patients, 18.9% (n=7) either could not remember or reported a conflicting decision that was recorded in the GP notes.  HCPs and patients agreed on the level of patient involvement in decision-making in 43% (n=16) of consultations.  70% (n=26) of GPs reported decisions were shared, however, 48.6% (n=18) of patients reported the decisions were shared in the same consultations. GPs reported that the involvement in SDM in the consultations were: GP driven - 22% [n=8]: Shared - 70% [n=26]; Patient driven - 8% [n=3]. Older patients reported that the involvement in SDM in the consultations were: GP driven - 48.6% [n=18]: Shared - 48.6% [n=18]; Patient driven - 2.7% [n=1]. |
| Kempen et al. (2020) Sweden^80^ | Questions:  "Can you describe the decision-making process after the contact with the pharmacist?" Probes: "To what extent could you ask questions, concerns and problems?" "To what extent did the pharmacist listen to you?" "How clear were the answers to your questions?" "Can you describe the decision-making process after the contact with the pharmacist?" Probes: "Who took the decision about medication treatment?" "To what extent were you involved in the decision-making (did you get any information)?" "To what extent were your values, preferences and needs taken into consideration?" "Which information did you get about risks and benefits regarding different alternatives?" "To what extent were family or caregivers involved in decision-making about medication treatment?" "To what extent were you informed about changes during hospital stay?" "To what extent should you have wanted more (or less) information? | “I sure did have questions, but […] I did not really know what the conversation was about” (p. 154) “I agreed to try it out as she [the pharmacist] suggested” (p. 154) “The patient doesn't get involved, but I think it would be very useful to understand why you get this medication” (p. 154) “It is your own choice to refrain from certain medications and take the risk that something happens to you” (p.154) “We, the patients, didn't receive so much information before […] they just gave us our medication and you had to take it without questioning. But now […] you can question certain things and ask about it, which I think is very important” (p.154) “I only received this medication list. They said they had taken away one [medication], but which one, or why, I don't know. […] I don't like that, I think they should explain why and what could happen. […] If I wouldn't have received this list, I wouldn't have known which medications I had” (p.154) “If you're in hospital like this and just had a stroke, you're not in the right condition [to talk]” (p.154) |
| **Author (s), (Year), Country, Ref** | **Questions asked/Topic guide in study related to SDM** | **Descriptive Results / Supporting Participant Quotes** |
| Knight et al. (2013) UK^79^ | Topics: "The participant's experience of the hospital discharge process (planning and preparation for the discharge ⁄ continuing arrangements for medication once back in the community); their knowledge, management and understanding of the medicines; the wider context (who else may be involved in the medicines management process)" | The majority of patients (n=17/19) had a change made to their medications whilst an inpatient. "I think that because I would ask if I had to, if I wasn't happy, I would take it upon myself to ask. I think you have to be prepared to do that. I mean I would, but not everybody would. I mean I work with the doctors" (p.286) "Yes, yes. They gave me a list of what they'd prescribed. Interviewer: And did you feel quite happy? Yes, I don't think I take it in about these medicines they're there and I feel he's got to take them, as I said to you before… (Laughs) I'm a bit lax about that, not knowing what they're for" (p.286) "That was a bit of an issue really …. there was no card to give any explanation and no nurses gave any explanation about it" (p.286) "‘‘This is what you've got’’. Never a comparison with what you came in with and what you're going out with because quite often there were differences. No explanations of why and that" (p. 287) "Well they could have said some has been altered and it's now this, because they sent some different to mine" (p.187) "Yes they said 'doctor so and so decided you don't need those and has taken them away'" (p.287) "What one is that? You see this is another thing, nothing's been explained to me! I've never seen that one before in my life! It was in the bag when I came out of hospital!’’ (p.287) |
| Kreling et al. (2006) US^66^ | Questions: "Was chemotherapy offered as an option? What was said about it? What did you think would be the results of chemotherapy? Were there any side effects you were worried about? Did you consider not having chemotherapy? Where did you get the information about chemotherapy?" As a final question, participants were asked to complete the sentence. "As an older woman with breast cancer, when I thought about having chemotherapy, I considered the following: . . ." Women were also asked, "How is that different from what you would have considered when you were a younger woman? Do you think it would have been different if the doctor were a different gender?" | "Whatever the doctor tells me to do, I’ll do. If they tell me I need a very strong chemotherapy, then I’ll do it. I told my doctor, ‘I’ll do whatever you tell me" (p.1068) I was very glad because they wrote things down and they took notes. Afterwards I had totally blanked out on various things that were said" (p.1071) |
| Lansbury (2000) Australia^62^ | Questions:  "Have you had conflicting advice from (HCP), others ? Were you given sufficient facts, were you given the opportunity to discuss your condition, were you given a realistic picture? What types of pain management do you know about - which ones have you tried, why haven’ t you tried the others? Where have you found out about different pain management?" "Have you come across any barriers to your pain management?" | "Doctors just grunt and write out a prescription without telling you anything. They should hand us a leaflet with the information if they aren’t going to talk to us" (p.9) "They say you can refuse treatment. If you do refuse medication, you can’t, they kind of stand over you and you’ve got to take it. I didn’t have any say" (p.9) |
| Manias et al. (2024) Australia^50^ | Questions:  Have your medications changed since you first came to hospital? How have you been involved in decisions about changes to your medications when you first came into hospital? Have you transferred from another ward (or hospital)? If so, how have you been involved in decisions about changes to your medications before coming to this ward? How have you been involved in decisions about changes to your medication while in this ward?  How do you involve patients or their families in decisions about changes to medications when patients are admitted/discharged to your ward? | "Med6: I think we should change the morning dose of risperidone. Resident: Yes, the prescribed dose of risperidone is 0.25mg BD. Med6 to resident: Can we change that to a night dose starting tomorrow? Resident: Yes, I’ll make that change. Med6 to patient: We’re going to change risperidone from daytime to night to help you sleep and to keep you calm. Sound ok? We want you to be really awake during the day so that you sleep at night." (p. 525) "It’s more if the patients … ask for it rather than I would go out of my way to talk to everyone about their medicine changes." (p. 526) "I do not want to be more involved in decisions to change my medications … They [doctors] know what they are doing, because I don’t know what was going on" (p.526) "This morning the doctor said he’d have a heart doctor see me and maybe think about whether the medication needs changing … I’m taking a lot of diuretic at the moment … [and I have] a catheter, and I said to the doctor, “Well if that’s taken out I want the diuretic decreased because I have accidents going to the toilet … they listened … this morning, it’s usually two tablets but there was only one" (p.527) "I’m happy as long as I’ve got the information in hand. My need [to be involved] may have frightened some people, I can understand that, but I needed the information in hand so that I could make the decisions … A couple of days ago I said ‘No! I won’t go on the baclofen.’ And then I’ve thought about it and we’ve talked about it and the doctors came back again and we reflected on it and I thought ‘Well as long as they’re monitoring my level of confusion I’ll go on it’. (p.527) |
| McCabe et al. (2019) UK^28^ | Observing Patient Involvement in Decision Making scale (OPTION-5) (items rated 0-4 - score 0-100):  "rating whether the doctor (i) describes different treatment options; (ii) supports the patient in becoming informed; (iii) checks understanding of all reasonable options; (iv) supports patients to examine preferences; and (v) integrates patient preferences into the decision" Satisfaction with Decision Scale (SWD) (5-point Likert scale - score 6 - 30):  1. I am satisfied that I am adequately informed about the issues important to my decision 2 The decision I made was the best decision possible for me personally 3. I am satisfied that my decision was consistent with my personal values 4. I expect to successfully carry out (or continue to carry out) the decision I made 5. I am satisfied that this was my decision to make  6 I am satisfied with my decision Patient Experience Questionnaire (PEQ) (5-point Likert scale - score out of 5 each):  Communication experiences - "We had a good talk", "I felt reassured", "The doctor understood what was on my mind" and "I felt I was taken care of";  Communication barriers - "It was a bit difficult to connect with the doctor", "Too much time was spent on small talk", "It was a bit difficult to ask questions", "Important decisions were made over my head" Autonomy Preference Index (API) (5-point Likert scale - score 6 - 30): Not used in analysis 1 .The important medical decisions should be made by your doctor, not by you 2. You should go along with your doctor's advice even if you disagree with it 3. When hospitalized, you should not be making decisions about your own care 4. You should feel free to make decisions about everyday medical problems 5. If you were sick, as your illness became worse you would want your doctor to take greater control 6. You should decide how frequently you need a check-up | OPTION-5 (rescaled 1-100): mean 22.5 (SD 17.25)  Highest scoring OPTION-5 item - Item 3 (out of 4) "Check understanding of all options" mean 1.32 (SD 0.86) Lowest scoring OPTION-5 item - Item 2 (out of 4) "Establishing partnership" mean 0.34 (SD 0.69) |
| Mc Gillicuddy et al. (2019) Ireland^67^ | Topics: "Experiences of medicine administration and modification Decision-making around medicine modification Knowledge about medicine modification Views and knowledge about medicine formulations Strategies used to overcome difficulties with formulations Healthcare professional support and involvement Supports" | “We decided ourselves, as a family … that we would half a tablet because she is a very small woman and she'd been complaining, not complaining, but that she was constantly tired. So eventually we put two and two together and decided to reduce that to half a tablet … we just informed the doctor and he said that's fine” (p.1429) “Whatever the doctor prescribed she got, nothing else, good, bad or indifferent because we believe in, strongly, in doing what doctors tell us and that's it” (p.1430) “I'd like to reduce tablets. I was on more before … I'd love to have them reduced again but I mean if that's what they say, I take them” (p.1430) “I mean if you are in hospital they just give you the medication and say take it, they give you the tablets in the little container and they bring you in water and they maintain that you are going to take them like and that's it” (p.1430) “I don't ask and I think afterwards why didn't I ask him …” (p.1430) “I would question everything, I don't have a problem…I would ring the pharmacist if I was having a problem with a particular tablet, would there be an alternative?” (p.1431) |
| **Author (s), (Year), Country, Ref** | **Questions asked/Topic guide in study related to SDM** | **Descriptive Results / Supporting Participant Quotes** |
| Mecca et al. (2022) US^43^# | Transcripts assigned: "a) the number of participants who had a given recommendation made in the Tool to Reduce Inappropriate Medications (TRIM) [see Fried et al [36]) report;  b) the proportion of participants who had any discussion about the medication(s) involved in the recommendation;  c) the proportion of participants who had a change in the medication(s) involved in the recommendation" As well as: "similarities and differences in medication communication and decision making" | Descriptive: There were n=86 (intervention) and n=50 (control (with assessment)) TRIM recommendations made within the patient groups. Of these: Intervention: 49% (n=42) medications discussed; 2.3% (n=2) medications discontinued; 2.3% (n=2) plans to discontinue meds; 3.5% (n=3) doses decreased Control (with assessment): 16% (n=8) medications discussed; 2% (n=1) doses decreased Quotes: "Clinician: You’re on losartan 100. We could try dropping the losartan a little bit to 50 and see if it helps with that lightheaded feeling. Would you like to try that?" (p.2718) "Clinician: Omeprazole, one tablet, daily. And I think the reconciliation that they did up here said that you don’t take it very often? Patient: Right. Clinician: So you’re taking kind of once in a blue moon or … Patient: Yeah. Well if I have problems with indigestion or whatever, I use it for several weeks and then it alleviates that problem. Clinician: Okay, so, okay, so you’ll use it just for a couple weeks at a time? Patient: Yes. Clinician: Okay, that’s fine to use it that way" (p.2718) |
| O'Quinn et al. (2015) US^82^ | Topics: "barriers to, and facilitators of effective medication management, knowledge of medications, strategies to improve medication management and question asking during interactions with HCPs". | “[The doctor] whittled it down to a certain level of what she needed. When he got ﬁnished with her, [she was taking] maybe four or ﬁve pills ... I worked with them in that area so I did suggest that there were too many pills and they needed to be looked at, to see if she needed those” (p. 328) "They review it and they verbalize that they’re on a quest to look at these pills . . . and as a result of that, I’ve been able to get off one whole blood pressure pill a day, and we just titrated down. . . I admire that they’re looking at trying to eliminate unnecessary medications. You know they just need to review it and talk to you about it" (p.328)  “My mother-in-law is very soft spoken, and when she was going to the doctor, she always said: I feel like they don’t hear me, they don’t pay attention to what I say” (p. 330) “Maybe even somebody going into the doctor with somebody else and saying, you know you might think that your taking what you need, but if somebody else sat down and said ‘Well, what is he taking this for? What is she taking this for?” (p. 330) "[There needs to be a] healthcare advocate who’s not a doctor, a pharmacist or an insurance salesman . . . someone who knows their medicine. They aren’t going to treat you, they aren’t going to lie to you, because it doesn’t do them any good one way or the other. They only get paid to advise you!" (p.330) |
| Ouellet et al. (2022) Canada^72^ | Topics: Older adults: "prescribing and deprescribing, ... concerns and relationship with health professionals"  Carers: same as above, as well as the "decisions that caregivers must make: contexts, constraints and concerns, as well as how decisions are made or not made" HCPs:  "experiences with prescribing and deprescribing, the problems they posed, ... and the relationships and communications with the very old adult and caregivers" | "Maybe it’s because I trust my doctor, because I know that if she prescribes all this for me, it’s for my own good, so I agree with her" (Older adult, p.4) "I would never try that. I have never stopped taking medication on my own. No, if something bothered me, I would call and tell the doctor right away. I have to warn him. It could be dangerous not to" (Older adult, p.5) "I don’t question my doctor’s decisions. He decides what is best for me" (Older adult, p.5) "I rely solely on the doctor’s opinion. He is the one who knows. I never search for answers to medical questions on the Internet" (Older adult, p.5) "Patients 80 years and older still have the mentality of following doctor’s orders. The doctor has some authority, so if he prescribes medication, the patient will think—well he prescribed it, so I’m going to take it" (HCP, p. 5) "They don’t question doctors’ decisions. Nor do they necessarily question all the medications they are prescribed" (HCP, p.5)) |
| Parekh et al. (2019) UK^81^ | Topics: Interview:  Describing the discharge experience and the first few days at home. Prompts: information provided about changes; was understanding checked; ability to remember; information provision (e.g. leaflets), involvement in DM about meds, extent/opportunities to be involved, SDM Focus group:  How common are medication-related problems at discharge? What factors might contribute to this problem? Prompts: communication, knowledge, time, information provision, SDM | "When you’re in hospital and you get prescribed these pills they don’t ever tell you what they are and what they’re for… they discuss things themselves and the patient is not involved, which is wrong…it’s like a secret society" (p. 149) "I come out and they give me a pile of medicine to take which I didn’t know what to do with and I had to get somebody, don’t know what it was, come around and sort out medicine for me… that was that, and that’s for afternoon and two of them, you know, ‘cos I didn’t know" (p.149) "I suppose you could go to your doctor if you were worried to ask a few questions, but we’re the sort of people that, you know, ‘oh I’m afraid to go to the doctor’, you know, he’s too busy to mess about just answering silly questions like that’ (p.149) "Well you trust your doctor don’t you, I mean, you think they’re right and you take them" (p.149) "They’re telling you you know nothing, whereas you’re living it (medication problems) and you do know" (p.149) |
| Peat et al. (2023) UK^57^ | Questions: (Older person) I know you had one/a number of your regular medicines stopped by your HCP recently. Why did it happen, how and which medicine(s)? Whose decision was it to begin with? [If healthcare professional] Did you agree with the decision at the beginning? Did you feel like you had a say in this decision? [Alternative] Were you given the opportunity to fully discuss your thoughts and explore your options? Do you feel that you are involved in discussions and decisions about your medicines or do you usually defer decisions to your healthcare professional(s)? [if deferral] Why do you think this happens? And are you happy that decisions are deferred to your HCP? When making decisions about your medicines, do you often talk to your HCP about your personal care goals and priorities, particularly if you disagree with a decision? Does your HCP ask what your views are on the subject before making a decision? Have you ever disagreed with a decision made by your HCP about your medicines? [If so] Can you talk about it and what the outcome was?  Do you feel supported and listened to by your healthcare professional(s) when you have questions or concerns about your medicines? | "I think a lot of older patients like the more prescriptive approach from a practitioner; they like to be told what to do" (p.284) "I think they should … talk to you more about it, instead of just going in and saying, ‘now we’re stopping your medication’. And that was it, that’s all they said" (p.285) "They don’t’ explain" (p.286) "It was the patient’s daughter who rang on her behalf. The patient had been having some dizzy spells … and so I talked to her daughter about what we were going to do … we agreed that it would be sensible to try and stop one of her anti-hypertensive medications and see how she went with that" (p.287) "… but I tell them all my information really should go down to her (informal carer), which they should be telling her more" (p.287) |
| Perreira, Bieri, del Rio Carral et al. (2022) Switzerland^45^$ | Topics: Older person: "Experience with medication received in hospital: - Changes to usual treatment in hospital - Information received in hospital about medication changes - People involved" Joint older adult and informal caregiver: "Process of hospital discharge and return home: - Experience with medication changes - Information received" | "They could have talked to me about it before stopping it [medication]. (…) They’re all assistants, lots of them… There’s some big professor on each floor, the rest are all assistants with their computers on wheeled stands and they type as they walk along. That’s it, eh. They don’t say much (…). They don’t communicate with anyone. They talk among themselves. They come in together; they leave together" (p.6) "So, there are some people who don’t worry about that. They… they swallow their pills any old way. No, me, I want to know what I’m taking. And why. (…) So, I don’t know why they change my medication without telling me. They just give you the prescription, and that’s it. No, me, I want to know the whys and wherefores” (p.7) “He’s a very agreeable patient, because he’ll never contradict, never… He lets himself be cared for. ... He’s brave, but he’s passive too. That’s to say, he may get annoyed inside, but he doesn’t show it. I can’t do it, but he listens to what he’s told and then he does what he’s told. He accepts it" (p.8) "They did what they were supposed to, huh. I couldn’t say, ‘No, I don’t want those particular pills.’ Why? Because I was in their hands. I had to do what they wanted, you know?" (p.8) "she’s come to a stage where she doesn’t give a damn. (…) She’s arrived at a stage in her life where she tells herself, ‘Well, I don’t have any choice, do I?" (p.8) |
| Perreira, Bieri, Martins et al. (2022) Switzerland^44^$ | Topics: Older person: "Experience with medication received in hospital: - Changes to usual treatment in hospital - Information received in hospital about medication changes - People involved" Joint older adult and informal caregiver: "Process of hospital discharge and return home: - Experience with medication changes - Information received" | “So, I said to myself, I’ll keep taking the [Atorvastatin®]. But as soon as I get the stent—in a little while—I’ll tell the doctor, ‘Well, I can stop that now.’ You see, I’m anti-medication” (p.411)  “But over there, you’ve no, you’ve got no say in it. They do stuff and that’s the way it is. That’s the problem. (...) I simply obeyed” (p.411) "She was very careful. Whereas now, she has reached a stage where she doesn’t care. I think that she has reached a stage in her life where she says to herself, ‘Well, I have no choice. I can’t manage anything anymore'" (p.413) “They [healthcare professionals] know more about drugs than I do” (p.413) "We know nothing. We can't keep up ..." (p.413) |
| **Author (s), (Year), Country, Ref** | **Questions asked/Topic guide in study related to SDM** | **Descriptive Results / Supporting Participant Quotes** |
| Reeve et al. (2016) Australia^73^ | Topics:  Beliefs about medications, "medication cessation, and what would make them more or less likely to consider cessation or be willing to cease based on a recommendation" | "I took it and had severe side effects, mainly dizzy, it was clearly not suitable for me and it was immediately apparent and I stopped taking it" (p.e555) "It would be good [stopping a medication] but you would have to have a discussion about it" (p.e555) "I think that’s the key thing, communicating to understand why something is going to be stopped, the reason for it and the effect it might have" (p.e555) "He was sick of taking all this medication … the doctor said to him, “Don’t do that, you’ll end up like my father who’s gone had a stroke and he’s just now like a vegetable”, so he kept on taking the medicine" (p.e555-6) "Every time I ask for a script for the things that I need … he [GP] must in his own mind review the need for them and not that we discuss that but I imagine that … he would be thinking yes will I or won’t I, and then he decides to write the script and that sort of review is good enough for me and I will happily continue to take the two that I need" (p.e556) "Facilitator: 'Are there any fears that you have about stopping medicine?’ Participant: ‘Not if I’m told to do so by someone [the GP] who knows better than me’" (p.e557) "One month they [a friend] spend $300 on medication … and I said what are you taking, and so she showed me some, and I said reduce it, go to your doctor ask to be reduced" (p.e557) |
| Ross & Gillett (2021) Canada^74^ | Not reported | "You have to trust whoever is making decisions for you." "I don’t think I’ve ever said no to any med" (p.2589) "Well, I take my medication because my doctor told me to take it … they know more than I do, so I would obey them and take the medication" (p.2590) "I’ll just wait and see what [my doctor] says and go with it, whatever she says because I’m assuming that she knows what she’s talking about. That’s all I can do" (p.2590) "If they keep me healthy, I’ll just listen to my doctor and keep on taking them" (p.2590) "So you just accept that they think it’s better for you to take fewer. And just, I have my faith in the medication, the doctors. You just do" (p.2592) "I know the people who were recommending different meds and so on at the hospital. I didn’t know those people, didn’t know their background. They didn’t know me. Whereas my cardiologist, I’ve had her for a number of years. She knows my background. She knows what’s happened to me, so I trust her more than some stranger" (p.2592) |
| Sale et al. (2011) Canada^75^ | Questions: "What recommendations did you receive by your health care provider(s) regarding your bone health after your fracture? Probe: What about the medication that was prescribed to you?" "What are you doing about those recommendations?" "What motivates you to take/not take your osteoporosis medication? Probe: What makes it difficult?" "What (or who) made you decide to take/not take your medication? Explain" | “expertise and judgment and follow[ed] whatever recommendations he [was] given without question” (p.4) “came from an age where people [did] what they were told” (p. 4) “he [specialist] didn’t say anything that convinced me that I needed to take the medication” (p.4) “If she [specialist] had said to me, I want you to drink three gallons of orange juice a day, I probably would have said, okay” (p5) "I thought, he’s a doctor. He knows what he’s talking about. If it’s going to help me, what am I going to do? Just let them sit on that dresser? That would be silly. Take them and try to get better” (p.4) ""her GP as “an excellent GP” who gave “advice that she thought was best for [her]” but it was her decision to “reject that advice”" (p. 6) |
| Salter et al. (2014) UK^83^ | Topics: "Current usage of preventive medicine, motivators and detractors from taking medication, and follow up with healthcare professionals" | Descriptive: 30% (n=9) of participants reported making the decision to not take bisphosphonates without discussion with their HCP.  Quotes: "He was quite happy, he said alright just stop. He said we’ve had no broken bones in your family, he said you’ll probably be quite alright" (p.3) "There is no bone disease or any sign of it in our family but I said I would take it and see what happened. It didn’t suit me and I said I’m not taking them anymore and he (doctor) agreed. He said ‘it’s no good taking them if they upset you because your diabetes is more important’" (p.6) |
| Schmittdiel et al. (2010) US^85^ | Questions: "Thought the issue of prescription drug cost was important enough to raise with their doctor" "Wanted their doctor to consider the cost to them when choosing medication"  "Talked with any doctor about the amount they had to pay for prescription drugs" "Asked if their doctor switched any prescriptions to a less expensive medication because of cost, or if they had used any medication less often than the doctor prescribed due to the amount they had to pay" | 44% (n=642) of patients reported talking to HCP about the cost of medicines.  76% (n=1108) of patients wanted to talk to their HCP about the cost of medicines. "Patients aged 65-74 years were more likely to discuss prescription drug costs with their HCP than those over 75 years (50% vs. 41%, p < 0.001)" (p.3) |
| Schopf et al. (2018) Germany^68^ | Topics: "The active role of patients in communication with focus on important topics associated with polypharmacy; ... what ... might help patients to engage actively during GP appointments" Questions: "Can you think of anything else that could help you or other patients address things in the doctor consultation, particularly if it is about tablets?"  "And do you think it is helpful if you have a bit of information beforehand, before you go to the doctor? "And could you perhaps think about something to motivate patients to address it anyway or what could help them?" | "she is "…only the patient and he is the doctor, the boss"" (p.358) "he’s always looking it up, he has it in the computer…but he has never asked whether I also take it" (p.358) "Then you just take it. As you behave: obedient and (laughing)" (p.358) "I always assume that the medications are prescribed in such a way that the doctor knows what he is doing. I assume that he knows about the effect of the medications and he knows how the medications interact with each other. That he knows that. That the deliberation regarding me already took place in the background. So I do not ask for that at all" (p. 359) "I know that from my daughters, who say: ‘Now you are going to the doctor and also tell him that’ and so on. Perhaps that is less the case with some people who are totally alone or so on. So, one sometimes needs a bit of a push" (p.360) "Depending on what it is about, one of the daughters comes with me. She will probably talk more than me. Because one probably talks more openly at home after all or in my case I forget it again and think: ‘Oh dear! You wanted to say that as well’. I have to start with making notes. That and what I want to know" (p.360) |
| Smith et al. (1994) US^60^ | Questions: "The doctor explains 22. My doctor writes down what I need to know about a medicine 37. The doctor explained fully the benefits of the medicine prescribed 38. The doctor explained fully the risks of the medicine prescribed The doctor encourages patient talk 15. My doctor asks my opinion about the medicine 19. My doctor encourages me to ask questions 23. My doctor waits for me to tell him/her what is on my mind 24. My doctor sets time aside for me to ask questions The doctor shares decisions 16. My doctor tells me about several medicines that might help me and asks which I prefer 35. At my last visit the doctor asked me to choose a treatment for my health problem 36. The doctor gave me enough information to make my own decision about which treatment to choose" | Descriptive:  The doctor explains 22. Mean 3.6 SD 1.9 37. Mean 4.5 SD 1.8 38. Mean 3.6 SD 2.0 The doctor encourages patient talk 15. Mean 3.8 SD 1.8 19. Mean 4.6 SD 1.5 23. Mean 4.8 SD 1.4 24. Mean 4.4 SD 1.7 The doctor shares decisions 16. Mean 3.0 SD 1.8 35. Mean 2.2 SD 1.7 36. Mean 2.7 SD 2.0  Comparisons of items using *paired t tests*: HCPs are forthcoming about the benefits (Item 37) of medicines, not so much on the risks (Item 38): *t*(154) = 6.3, p<0.001 (significant). HCPs will ask patients questions about medicines (Item 15), they explain, they encourage their patients to engage in the conversation and ask questions (Item 19, 23 & 24), however they do not appear to involve patients in medication-related decisions (Items 16, 35 & 36) - Comparing Item 15 (lowest doctor encourages) with Item 16 (highest DM): *t*(175) = 6.22, p <0.001 (significant). |
| **Author (s), (Year), Country, Ref** | **Questions asked/Topic guide in study related to SDM** | **Descriptive Results / Supporting Participant Quotes** |
| Spinewine et al. (2005) Belgium^76^ | Topics & Questions: HCPs: "Prescription and follow-up; transfer of information, counselling" "Which difficulties do you face in the prescription of medicines (/follow-up) for elderly patients admitted on your unit?" Older person: "knowledge of treatment, treatment changes, information received, difficulties encountered" "Let’s talk about the changes that have been made during your stay here with regard to your medicines." Probes: "What makes you happy about it? What makes you unhappy? What bothers you? Which difficulties have you encountered? Tell me about the last time somebody told you something about your medicines" | "I think that if somebody explains to the patient why he or she is given this medicine, the patient could understand. Here patients mightn’t understand, quite simply because they [doctors or nurses] don’t know how to explain things to them" (HCP, p.3) "I think that too often, they don’t ask what the patient thinks" (HCP, p.3) "The doctors tell me, “We’ll stop this one and give you something else that will work better.” Well, for me that’s fine. I have boundless confidence in them" (patient, p.3) "I’m completely lost . . . My medicines were replaced by different ones, but I don’t know who decided that . . . and I don’t know what they are. . . . I would like to know what I’m taking and what I am being treated for. I like to know the “why” (patient, p.3) |
| Thevelin et al. (2022) Europe^49^ | Questions: "During your hospitalization, the following medication changes were proposed (remind the changes). Could you tell me how these medication changes were proposed to you?"  "When deciding to change a medication, there are 3 possible ways to proceed. It is either the doctor that decides alone, or it is the patient that takes the decision alone or it is a shared decision. How was the decision of changing your medication taken during your hospitalisation?" Prompts: "Was there something that helped you in deciding on medication changes? Was one of your family members or a carer involved in the discussion? If yes: did they help you to make decisions on your treatment? If no: would you have preferred someone to be present? To what extent were you satisfied or not with your involvement in decision-making? Would you have liked to participate more? Not participate? If the patient did not participate: what kept you from being involved in the decision? In an ideal world, how would you have liked that the decision making on medication changes occurred? How do you see your role as a patient in making decisions about your medications?" SDM-Q-DOC (SDM questionnaire for Doctors) (6-point Likert scale - score 0 to 100):  (1) I made clear to my patient a decision needs to be made. (2) I wanted to know exactly from my patient how he/she wants to be involved in making a decision. (3) I told my patient that there are different options for treating his/her medical condition. (4) I precisely explained the advantages and disadvantages of the treatment options to my patient. (5) I helped my patient understand all the information. (6) I asked my patient which treatment option he/she prefers. (7) My patient and I thoroughly weighed up the different treatment options. (8) My patient and I selected a treatment option together. (9) My patient and I reached an agreement on how to proceed. | Quotes (Paper): "No-one explained anything to me! When I was discharged they just told me, so you’ve got this and that, and this instead of that. And that’s all. As for the whys and wherefores, I’ve no idea" (p.891) "And when you start asking why, sometimes I think they find it hard to explain things. They all have their drug lingo. And that’s what’s difficult to grasp at times" (p.891) "They [the doctors] would just come along in a hurry, they come and go and that’s it, it’s done" (Patient, p.891) "You don’t get a say in the matter, do you? When it comes down to it, all you have to do is swallow what they put in your mouth" (p.892) "I made the decision [decision to not commence a statin proposed" p.892) “You go to the doctor to be healed, not to discuss preferences” (p.892) “In hospital you just take medications, you don’t ask questions” (p.892) "I assume the doctor knows more about it than I do, so I have to accept it" (p.892) "So if my grandson hadn't intervened, maybe they wouldn’t have given me Lyrica and wouldn’t have discussed things with me more" (p.893) "I trusted them blindly" (p.893) Quotes (Supplement 6):  "If you don’t ask about it [for information about medication changes], they don’t tell you anything" "No-one explained anything to me! When I was discharged they just told me, so you’ve got this and that, and this instead of that. And that’s all… As for the whys and wherefores, I’ve no idea" "But they just said “we’ll do this, this and this” and they’d be gone!" "That wasn’t discussed with me [stop medications]. I suddenly realised, hey, I’m not being given those [medications] anymore" "So it’s up to me to take them. That means I decide myself. I could refuse to take them, after all. I would have the right to do that. So they just proposed them to me and then it would be up to me...// Yes, actually up to me" "I hadn’t [the desire to ask] because they completely ignored me. As if I wasn’t there at all. I thought they should have discussed it [decision to commence opioids] with me because I was the person taking them" "I just take what the doctors prescribe, and I do so consistently. It mightn’t taste great, but I take them" "I take it because it’s prescribed, and that’s that" SDM-Q-DOC: median 76 (range 69-82) Compared to the SDM-Q-DOC, 23% (n=11) of patients reported involvement in DM. Of the patients from the intervention arm of the main trial study (n=27); 30% (n=8) reported involvement in DM, however, it was reported by the research assistant in the main study that 70% (n=19) patients were observed to be involved with SDM according to the trial's criteria. |
| Tietbohl & Bergen (2022) US^77^ | Transcripts assessed for: Patients asking questions "with a specific type of action projection" (agency framing). | Quote in relation to HCP's advice (in brackets) on patient making a decision:  "Patient: Oh, and then last time. You told me that old people shouldn't take a lot of chlorzoxazone. And I wanted to ask you what's a lot? Doctor: Heh hem how much are you taking? Patient: Well since you told me that six months ago, I always break the pills in half. I probably haven't taken more than ten pills. Doctor: So th' daily basis is the hard thing. Patient: Oh, I see. Doctor: So you gotta let your kidneys and everything recover a little bit. Okay? Patient: Oh, I why, what does it do? Doctor: Oh it affects the pathway for kidney function. It chemically blocks mhm. Patient: It does? Thuh, those muscle relaxants do that? Doctor: Mm. Patient: Oh, I wondered what. Doctor: And then you can be dizzy, and unstable on your feet, so. Patient: Oh, yeah, no I really stopped. Doctor: Okay good. Patient: I did" (p. 5) Quote of patient deferring to HCP decision:  "Doctor: Suh your B12 is pretty low normal. Patient: Oh yeah, that's the other thing I wanted to talk to about. Those, those results. Doctor: Mmhm, Mmhm. Patient: What uh, that one that they highlighted ju know that got me contacting you, and I forgot to re-look it up when I was coming in. I everything eugh, I got the feeling that fru, I mean I know from your response that you didn't think there was a problem. Doctor: No, I'd like to give you a B12 shot now, n' then every time you come see me we'll do one. And then you can take B12 over the counter if you want. hhh. It's normal, but it's low normal. I like high, coz it can't hurt and it might help. Patient: That's fine with me, I'll go get B12 , that's no problem" (p. 7) |
| Tinetti et al. (2024) US^61^ | Topics and Questions: CollaboRATE (score range, 0-100; higher score indicates greater perceived shared decision-making and goal ascertainment): How much effort was made to help you understand your health issues? How much effort was made to listen to the things that matter most to you about your health issues? How much effort was made to include what matters most to you in choosing what to do next? Accountable Care Organization (ACO) shared prescribing decision-making quality measure (a higher percentage reflects better outcomes): “When starting a new medication, did your provider ask what you thought was best for you?” | Baseline follow up Differences   CollaboRATE Top Score Intervention Usual Care OR (95% CI) P Value Baseline 46.3 (36.5-56.5) 47.7 (37.9-57.8) 0.90 (0.50-1.70) .84 Follow-up 58.5 (45.6-70.3) 61.9 (50.9-71.8) 0.90 (0.40-1.80) .69  ACO Intervention Usual Care OR (95% CI) P Value Baseline 48.1 (38.2-58.2) 50.3 (40.4-60.2) 0.90 (0.50-1.60) .75 Follow-up 72.5 (56.4-84.3) 52.1 (36.7-67.2) 2.40 (0.90-6.40) .07 |
| Tinetti et al. (2019) US^47^ | Topics and Questions: Older Patient Assessment of Chronic Illness Care (O-PACIC) (11 items; range 1-5; higher score indicates better perceived experience of chronic disease care): Given choices on treatment to think about; Asked to talk about any problems with their medicines or their effects; Asked to talk about their goals in caring for their illness CollaboRATE (score range, 0-100; higher score indicates greater perceived shared decision-making and goal ascertainment): How much effort was made to help you understand your health issues? How much effort was made to listen to the things that matter most to you about your health issues? How much effort was made to include what matters most to you in choosing what to do next? | O-PACIC score Intervention mean 2.8 (SD 1.0); Usual Care mean 2.9 (SD 1.0) CollaboRATE score Intervention mean 84.2 (SD 19.3); Usual Care mean 83.5 (SD 23.7)  Baseline follow up Differences  Least Square mean (SE) Baseline - Follow-up  Intervention Usual Care Difference (SE) P Value O-PACIC −0.2 (0.2) 0.1 (0.2) −0.06 (0.1) .60 CollaboRATE −1.2 (5.3) 2.9 (5.2) −4.1 (2.8) .14  Changes similar across both groups. High at baseline and showed little improvement in change. |
| **Author (s), (Year), Country, Ref** | **Questions asked/Topic guide in study related to SDM** | **Descriptive Results / Supporting Participant Quotes** |
| Tjia et al. (2008) US^69^ | Topics: "(i) a self-assessment of comorbidities, (ii) communication with their physicians about medications, (iii) medication use" Questions: "Do you speak with your physician about your medications? If so, what specifically do you talk about?" "Is there anything else about your medications that you would like your physician to discuss during your visits?"  "Do you speak with your physician about the cost of your medications?" "Do you speak with your physician about the number of pills you take?" | "I don’t talk to my doctor about my medications because half of the time these doctors today do not have time to sit and talk with you. Not just my doctor, any of them do not have the time. I think they are limited to a certain time as to how long they are supposed to talk to a patient and visit a patient. And if they go over their time it’s like a crime, so I really do not have the time to talk to him about it" (p. 43) "Is there any new medicine came in that would help me more than what I’m taking?" (p.44) "There’s so many of them; when can we start cutting them down?" (p.44) "Is this going to be compatible with everything else I’m taking?" (p.44) “I see this doctor like every six weeks, Dr. (name) and he’s very nice to talk to. But the only thing I don’t do is take the medication he prescribes for me and I’m being very honest with you. If I can see where it’s doing me some good then I will take it” (p. 45) "... the doctor said don’t take that anymore, so I stop" (p. 45) "If they want me to take it, I will" (p. 46) "... I just say, well when she says I have to take medication, I hit the ceiling, you know ... but she discussed with me what each is for ... and I said another pill?" (p. 46) "my nurse practitioner told me that my numbers were good. I went back to the heart doctor and I gave him the message and he said 'Well I’m going to put you on [medication name] anyhow.' And I was thinking if I’m doing pretty good ... then why am I taking extra medicine? Because I just don’t want to be ..." (p.47) "Well I don’t, you know, I never question my doctor. If I go to him, okay, when I go to him and then he checks me out, he says like everything, he said you’re doing fine, I never question him ... I never come to conclude that I should question him about my medication ... because he is my doctor" (p. 47-8) |
| Tobiano et al. (2021) Australia^27^ | Phase 1 Audio recordings / Survey: "Preferred role in discussing medications with health care professionals: I like the health care professional and I to have a shared role in discussions about my medications I like the health care professionals to lead discussions about my medications I like to lead all discussions about my medications" To analyse audio recordings: "Thompson's continuum of patient participation" was used Questions: "What things helped you to get involved in talking about your discharge medicines?  What did you talk about with each type of health care professionals?  What makes it challenging for you to get involved in talking about your medicines prior to hospital discharge?  What was your role been in talking about your medicines before hospital discharge? Did you participate in medication communication the way you wanted to?  What was your family/friend/significant others’ role in discussing your medicines before discharge?" | Descriptive: 63.2% (n=24) patients and 33.3% (n=1) family members preferred a shared role in discussions about medications.  23.7% (n=9) patients and 66.7% (n=2) family members preferred the HCP to lead discussions about medications. 13.2% (n=5) patients preferred to lead discussions about medications.  Observations: Using "continuum of patient participation" across 71 encounters (n=43 pharmacists; n=26 nurses; n=2 doctors) Non-involved: n=21  Information-seeking: n=34 Information-giving: n=41 SDM: n=24 Autonomous DM: n=2 Quotes: "They (doctors) were reluctant to give her a pill initially, they wanted to give her an injection which would have meant twice a day … but I can't do that I'm sorry, so they went away and rejigged it all and said that there's this new Apixaban out and it might be a good idea" (Family, p.903)  "(Patient name) is very old-time … it's yes sir, no sir, 3 bags full sir when she's talking to doctors and she's always very deferent … whereas me, I'll say right-o chap … I like to know what's happening, but it's not always easy to get it. I will phone and say can I speak to (doctor's name) treating doctor and they say oh we can't find him, he's off somewhere else … "(Family, p.903) |
| Weir et al. (2021) Australia^54^ | Topics: "GPs’ views on the role of patients’ goals and preferences in medicines management including deprescribing" "GPs were asked about if/how they communicate with older patients about polypharmacy" | “the patients, um, have to believe their doctors but that belief is based on how well you’re communicating all their medical problems with them” (p.5) “it is sometimes difficult to marry up patient goals and clinician goals. Um, and I think that is due to health literacy as well and how well we explain things to patients when we start them on medications” (p.5) “I think it is very important. Because, their goals need to be somewhat aligned to ours for them to be compliant” (p.5) “A lot of patients … they’re quite happy to do what you say” (p.5) "I use a larger screen, a curved screen … [to] get patients involved, um, have some transparency … empower them with the fact that this is their health and they need to take control of that. So first getting them engaged and then having some control” (p.6)  “making sure that what we’re doing is in line with what they want. So ultimately, we’re there to improve [the patient’s] health, as they see it … not to treat the numbers” (p.6) “My interest mainly is quality of life for patients and asking them what … what that means to them. Taking medication for what their goals are” (p.7) “I want to say it’s a shared role. It’s not me telling you and you just listening to what I’m saying. I want you to interact. I want you to understand. I want you to challenge” (p.6) “I’m not sure that I really ask them a lot” (p.7) “That age group usually are happy to be guided by me. You know, they just take … whatever I say they just believe” (p.8) |
| Weir et al. (2018) Australia^46^ | Questions: Control Preference Scale: (participants chose the statement that best described their preference) "a) I prefer to make the decision about which medication I will receive, b) I prefer to make the final decision about my medication after seriously considering my doctor’s opinion, c) I prefer that my doctor and I share responsibility for deciding which medication is best for me, d) I prefer that my doctor makes the final decision about which medication will be used, but seriously considers my opinion, and e) I prefer to leave all decisions regarding medication to my doctor" Interview: Then asked their reasoning behind their answer.  “Can you think back to a discussion with your GP about your medications. What was that conversation like for you?” | Control Preference Scale: 30% (n=9) older adults reported a patient involved preference for DM (Statements a, b, c) 70% (n=21) older adults reported a preference toward HCP-driven DM (Statements d, e) Quotes: “I started coughing. All day. And I went to my GP and he said, oh yes, well we better change that. So he took that away and…gave me a different [one]” (p.e101) “Well, um, he knows best. He knows my condition. I’ve been with him for 20 odd years. So he knows me inside and out sort of thing” (p.e101) “It’s very hard for an 82 year old person to argue with doctors. They um can be very overbearing some GPs you know” (p.e101-2) “I have complete faith in her and I would never go against if she said something didn’t suit me” (p.e102) “I wasn’t happy taking them at all … I couldn’t function at all … not properly. So I did. I stopped them” (p.e102) “No, I don’t tell her because she’d say take it” (p.e102) “So I took myself off it. I’ve told doctor about it. I’m taking myself off it, and she said, we’ll see how you go” (p.e102) “I think they know better. I know better how I feel with it. But they probably know what better to … to prescribe for me” (p.102) “I feel I’m doing the right thing by the doctor”; “Well, he’s the one that makes the decision" (p.e103) “Oh, I don’t make any decision. It’s up to the doc … my local GP. I rely on him to do all that for me” (p.e103) “I have no basis from where to converse about it because I have no knowledge…absolutely no knowledge about them” (p.e103) |
| **Author (s), (Year), Country, Ref** | **Questions asked/Topic guide in study related to SDM** | **Descriptive Results / Supporting Participant Quotes** |
| Wilson et al. (2007) US^84^ | Questions: "Physician–patient dialogue items (response options were yes and no).  1. During the last 12 months, did your personal doctor talk with you about all of the different medicines you are using, including medicines prescribed by other doctors?  2. During the last 12 months, did you talk with any of your doctors about the cost of your prescription medicines?  3. During the last 12 months, did you talk with any of your doctors about changing one of your prescription medicines because the medicine was making you feel worse or was not working?  4. During the last 12 months, did any of your doctors switch you from one prescription medicine to a different one that would cost you less?" | Descriptive: 68.2% of all participants had a discussion about their medications with their HCP in the previous 12 months 30.9% of all participants had discussed medication cost with their HCP 28.9% of all participants talked about medicines in relation to adverse effects / efficacy with their HCP  27% of older adults who stopped a medication due to adverse effects had not discussed this with their HCP. 39% of older adults who did not take a medication due to cost had not discussed this with their HCP.  Subset of sample - Participants with 3 or more chronic conditions (n=5,739): 75.8% had a discussion about their medications with their HCP in the previous 12 months 41.3% had discussed medication cost with their HCP 41.9% talked about medicines in relation to adverse effects / efficacy with their HCP |
| Xu et al. (2003) US^48^ | Questions: “In the past 12 months, has your physician ever asked you whether you can afford a drug?” physicians’ Participatory Decision-Making (PDM) style: (5-point Likert scale 0 (never) to 4 (very often) - score out of 12) 1. If there were a choice between treatments, how often would your doctor ask you to help make the decision?  2. How often does your doctor give you some control over your treatment?  3. How often does your doctor ask you to take some of the responsibility for your treatment? | Descriptive: 11.36% (n=268) respondents reported that their HCP asked about medication cost.  According to age and reporting yes:  65–70 years 12.26%  71–75 years 12.25%  76–80 years 9.76%  ≥81 years 9.03%   PDM score (total sample): mean 6.66 (SD 3.57) Multivariate analysis, the PDM was a significant predictor of communication about cost (OR 1.070, CI 1.024 to 1.119, p<0.01). |
| * Articles reporting the same study # Articles reporting the same study | | Abbreviations: HCP -healthcare provider; GP - General Practitioner; SDM - Shared decision-making; DM - Decision-making; ED - Emergency Department; OR - odds ratio; CI - confidence interval; SD: standard deviation |
| $ Articles reporting the same study | |  |
| ⱡ Only excerpts from participants aged ≥65 years used | |  |
